# Parasubthalamic Glutamatergic Neurons Coordinate Cardiovascular Homeostasis and Locomotion in Mice

**DOI:** 10.1002/advs.202417353

**Published:** 2025-07-28

**Authors:** Ming‐Xuan Lu, Jin‐Yin Huang, Xing‐Zhe Xu, Ji‐Yu Sun, Dan‐Ni Zou, Jia‐Yao Zhang, Chen Chi, Qi Zhang, Wei‐Cai Liu

**Affiliations:** ^1^ Shanghai Engineering Research Center of Tooth Restoration and Regeneration & Tongji Research Institute of Stomatology and Department of Prosthodontics & Stomatological Hospital and Dental School & Tongji University 3663 Zhongshan North Road Shanghai 200072 China; ^2^ Shanghai Key Laboratory of Brain Functional Genomics (Ministry of Education) & Affiliated Mental Health Center (ECNU) & School of Psychology and Cognitive Science & East China Normal University Shanghai 200062 China; ^3^ Department of Cardiology & Shanghai Chest Hospital & School of Medicine & Shanghai Jiao Tong University Shanghai 200030 China

**Keywords:** cardiovascular homeostasis, locomotion, neural circuits, parasubthalamic nucleus

## Abstract

Cardiovascular homeostasis and locomotion are vital for animal survival and the maintenance of essential physiological functions. The parasubthalamic nucleus (PSTN) has recently emerged as a novel interoceptive center in the central nervous system, yet its role in coordinating cardiovascular homeostasis and locomotor behavior remains poorly understood. Here, we demonstrate that PSTN*^Vglut2^* neurons serve as a novel baroreflex center, exhibiting heightened activity during acute hypertension. Through their glutamatergic monosynaptic descending projections to the nucleus of the solitary tract (NTS), these neurons mediate parasympathetic output to regulate cardiovascular homeostasis while coordinately modulating locomotor activity. Chemogenetic activation of NTS‐projecting PSTN*^Vglut2^* neurons rapidly reduces heart rate, attenuates hypertensive responses, and accelerates blood pressure recovery. Conversely, their inhibition exacerbates blood pressure elevations, delays recovery to baseline, and impairs locomotor performance. Correlation analyses reveal a significant negative relationship between blood pressure fluctuations and locomotor metrics, such as total running distance and high‐speed locomotion duration, underscoring the interdependence of cardiovascular stability and motor function. These findings position the PSTN as a critical central command hub, offering novel insights into the neural circuits governing cardiovascular homeostasis and locomotion, with potential implications for targeted neuromodulatory interventions in hypertension.

## Graphical abstract

PSTN*^Vglut2^* neurons function as a new central baroreflex hub via projections to the NTS, modulating parasympathetic cardiac output to maintain cardiovascular homeostasis while synchronously regulating locomotion. Blood pressure fluctuations negatively correlate with locomotor performance. Chemogenetic activation of PSTN*^Vglut2^* neurons lowers heart rate, buffers acute hypertensive responses, and accelerates pressure recovery, whereas inhibition exacerbates hypertension and impairs high‐intensity exercise.

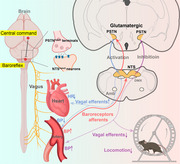

## 1 Introduction

Cardiovascular homeostasis is essential for maintaining normal physiological functions and supporting critical life activities. Increasing evidence reveals that locomotor exertion intrinsically challenges cardiovascular stability, creating an obligate interdependence between these systems.^[^ ^1^, ^2^, ^3^ ^]^ The brain continuously monitors dynamic cardiovascular states and regulates hemodynamics through mechanisms like the baroreflex,^[^ ^4^, ^5^, ^6^ ^]^ with bidirectional interactions being particularly vital during locomotion where metabolic demands escalate.^[^ ^7^ ^]^ Cardiovascular‐brain interactions, in both healthy and diseased states, is modulated by the ongoing interplay between the sympathetic and parasympathetic branches of the autonomic nervous system.^[^ ^6^, ^8^, ^9^, ^10^ ^]^ This autonomic regulation governs heart rhythm, blood pressure, cardiac output, peripheral immune responses,^[^ ^11^, ^12^ ^]^ and hematopoietic function,^[^ ^13^ ^]^ ensuring integrated homeostasis. The baroreceptor reflex exemplifies this coordination by providing real‐time cardiovascular stabilization, yet the central neural mechanisms enabling such adaptation remain elusive.

The nucleus of the solitary tract (NTS) is a pivotal hub within the parasympathetic nervous system, integrating baroreceptor afferents and visceral‐somatic signals to maintain cardiovascular function.^[^ ^14^, ^15^ ^]^ It orchestrates blood pressure and heart rate through the baroreflex,^[^ ^6^, ^16^ ^]^ mediated by parasympathetic efferent originating from the dorsal motor vagal nucleus (DMX) and ambiguous nucleus (Amb).^[^ ^6^, ^14^, ^17^, ^18^, ^19^ ^]^ Although brainstem/midbrain vagal pathways controlling cardiovascular responses^[^ ^8^, ^20^, ^21^ ^]^ and locomotion^[^ ^22^, ^23^, ^24^ ^]^ are well‐characterized, the fundamental mechanisms linking physical activity to cardiovascular regulation remain unclear. Higher brain centers modulate these systems via “central command”.^[^ ^5^, ^25^, ^26^, ^27^ ^]^ It remains unknown whether higher brain centers coordinately regulate locomotion and cardiovascular homeostasis. This underscores the persistent challenge of unraveling the neural circuits driving cardiovascular‐brain interactions.

The parasubthalamic nucleus (PSTN), a distinct vesicular glutamate transporter 2 (*Vglut2)*‐expressing nucleus, has recently been identified as a key node in sensing and regulating autonomic functions and homeostatic needs, including cardiovascular control, thermoregulation, feeding behavior, and aversive emotional processing.^[^ ^28^, ^29^, ^30^ ^]^ Positioned as a major upstream center in the hypothalamus for the NTS,^[^ ^31^, ^32^ ^]^ the PSTN exerts notable influence. For instance, PSTN stimulation in rats reduces mean blood pressure (MBP) and heart rate, likely through synaptic transmission within the NTS.^[^ ^33^, ^34^ ^]^ Additionally, fear‐induced activation of the PSTN triggers core hypothermia and tail vasodilation,^[^ ^35^ ^]^ underscoring its direct role in cardiovascular function. Based on this evidence, we propose that the PSTN acts as a novel central command center, processing ascending cardiovascular signals and executing descending regulatory actions. Elucidating the role of PSTN in orchestrating cardiovascular homeostasis and locomotion is therefore of substantial importance.

In this study, we sought to pinpoint specific neural circuits coordinating cardiovascular homeostasis and locomotion. We report that PSTN*^Vglut2^* neurons, via descending projections to the NTS, synergistically regulate cardiovascular homeostasis and locomotion. These findings may offer new possibilities for non‐pharmacological approaches to hypertension through targeted neuromodulation of this pathway.

## 2 Results

### 2.1 PSTN Neuron Ablation Impacts Cardiovascular Parameters and Locomotion

To investigate the role of PSTN neurons in regulating cardiovascular responses and locomotion, bilateral injections of kainic acid (KA) were administered to ablate PSTN neurons in C57BL/6J mice. Ablation efficacy was verified by NeuN immunostaining, which revealed a significant reduction in PSTN neurons three days post‐injection (Figure S1A–C, Supporting Information). Following a three‐day habituation period, baseline heart rate, systolic blood pressure (SBP), diastolic blood pressure (DBP), and MBP were measured. Voluntary running wheel adaptation training and formal testing commenced one day after blood pressure assessments (Figure S1D, Supporting Information). KA ablation did not significantly alter heart rate but significantly elevated SBP, DBP, and MBP (Figure S1E–H, Supporting Information). In a 12‐h voluntary running wheel experiment, KA‐ablated mice exhibited significantly reduced total running distance and maximum speed compared to controls, alongside increased immobility time (Vehicle: 63.52 ± 4.90% vs KA: 81.53 ± 5.90%) and decreased fast‐speed running time (Vehicle: 17.78 ± 3.96% vs KA: 5.16 ± 2.44%) (Figure S2A–D, Supporting Information). A 1‐h semi‐voluntary running wheel experiment corroborated these findings, showing reduced distance and speed, with a trend toward increased immobility (Vehicle: 4.67 ± 1.81% vs KA: 21.56 ± 8.14%) and reduced fast‐speed running time (Vehicle: 23.63 ± 2.32% vs KA: 13.04 ± 3.79%) (Figure S2E–H, Supporting Information). These results indicate that PSTN neurons play a critical role in maintaining cardiovascular homeostasis and supporting locomotion, particularly during high‐speed locomotion.

### 2.2 PSTN Neurons Coordinately Regulate Cardiovascular Function and Locomotor Behavior

**Figure 1:**
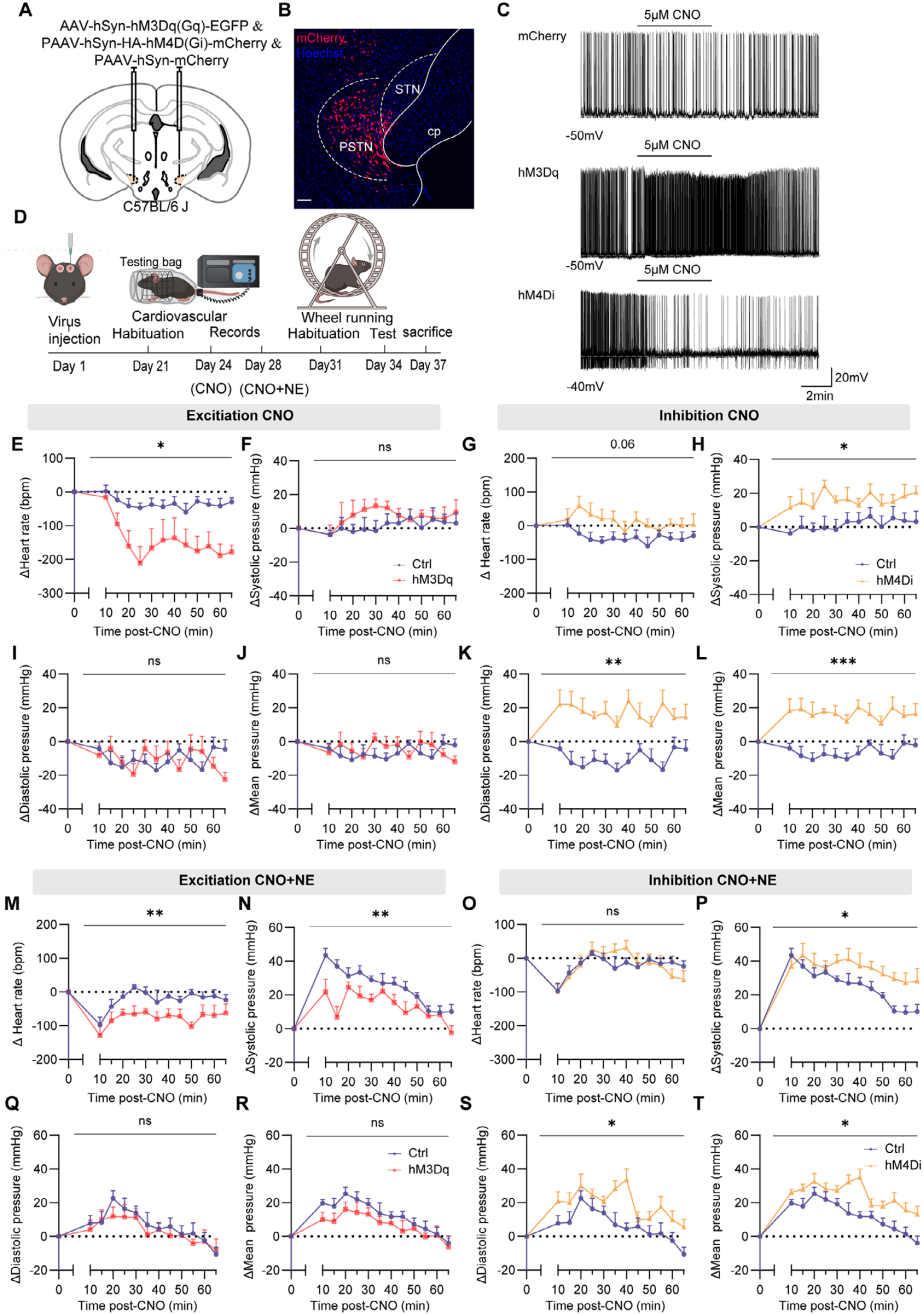
Chemogenetic manipulation of PSTN neurons alters heart rate and blood pressure. A) Schematic of microinjection into the PSTN of the C57BL/6J mice. B) Representative photomicrographs of the PSTN depicting mCherry expression from a C57BL/6J mice. PSTN: parasubthalamic nucleus. STN: subthalamic nucleus. cp: cerebral peduncle. Scale bar: 100 µm. C) Representative voltage traces recorded from an mCherry, hM3Dq or hM4Di ‐expressing during the application of CNO. D) Schematic of the protocol in experiments. E,F,I,J) Summary of changes in heart rate and blood pressure in C57BL/6J mice after administration of CNO, mCherry mice: n = 7; hM3Dq mice: n = 6. E) ∆Heart rate (Two‐way ANOVA F_1,11_ = 8.504, *p* = 0.014). F) ∆Systolic pressure I) ∆Diastolic pressure J) ∆Mean pressure (Two‐way ANOVA; not statistically significant). G,H K, L) Summary of changes in heart rate and blood pressure in C57BL/6J mice after administration of CNO, mCherry mice: n = 7; hM4Di mice: n = 6. G) ∆Heart rate (Two‐way ANOVA; not statistically significant). H) ∆Systolic pressure (Two‐way ANOVA F_1,11_ = 8.224, *p* = 0.0153). K) ∆Diastolic pressure (Two‐way ANOVA; F_1,11_ = 19.36, *p* = 0.0011). L) ∆Mean pressure (Two‐way ANOVA; F_1,11_ = 21.39, *p =* 0.0007). M,N,Q,R) Summary of changes in heart rate and blood pressure in C57BL/6J mice after administration of CNO and NE, mCherry mice: n = 7; hM3Dq mice: n = 7. M) ∆Heart rate (Two‐way ANOVA F_1,12_ = 12.42, *p* = 0.0042). N) ∆Systolic pressure (Two‐way ANOVA F_1,12_ = 14.30, *p* = 0.0026). Q) ∆Diastolic pressure (Two‐way ANOVA; not statistically significant). R) ∆Mean pressure (Two‐way ANOVA; not statistically significant). O,P,S,T) Summary of changes in heart rate and blood pressure in C57BL/6J mice after administration of CNO and NE, mCherry mice: n = 7; hM4Di mice: n = 7. O) ∆Heart rate (Two‐way ANOVA; not statistically significant). P) ∆Systolic pressure (Two‐way ANOVA F_1,12_ = 6.266, *p* = 0.0278). S) ∆Diastolic pressure (Two‐way ANOVA F_1,12_ = 6.055, *p* = 0.03). T) ∆Mean pressure (Two‐way ANOVA F_1,12_ = 9.255, *p* = 0.0102). NE, norepinephrine. Data are represented as mean ± SEM (E‐T), Δ represents the amount of change in cardiovascular parameters relative to baseline. Two‐way repeated measures ANOVA followed by Bonferroni post hoc test; ns, *p *> 0.05; *p < 0.05; **p < 0.01; and ***p < 0.001. See also Table S1 (Supporting Information).

To investigate the role of PSTN neurons in regulating cardiovascular function and locomotor behavior, we employed the designer receptors exclusively activated by designer drugs (DREADDs) system for targeted manipulation of PSTN activity. C57BL/6J mice received bilateral PSTN injections of AAV‐hSyn‐hM4D(Gi)‐mCherry (PSTN‐hM4Di), AAV‐hSyn‐hM3D(Gq)‐EGFP (PSTN‐hM3Dq), or control AAV‐hSyn‐mCherry (PSTN‐EGFP) (**Figure** **1**A). Imaging of coronal sections confirmed precise viral expression within the PSTN (Figure 1B). To validate DREADD efficacy ex vivo, whole‐cell current‐clamp recordings in acute PSTN slices demonstrated that clozapine‐N‐oxide (CNO, 10 µm, 6 min) significantly reduced spontaneous action potential frequency in hM4Di‐expressing neurons and increased spontaneous action potential frequency in hM3Dq‐expressing neurons (Figure 1C).

Following a three‐day habituation period, mice in all groups received an intraperitoneal injection of clozapine‐N‐oxide (CNO, 1 mg kg^−1^) and were gently guided into a testing bag. Heart rate and blood pressure were recorded 5–10 min after entry (10 min post‐CNO injection), as outlined in the experimental procedures (Figure 1D). In PSTN‐hM3Dq mice, CNO induced a rapid and sustained reduction in heart rate over the first 20 min, with partial recovery but persistently lower rates compared to PSTN‐EGFP controls throughout the recording period (Figure 1E). No significant differences in systolic blood pressure (SBP), diastolic blood pressure (DBP), or mean blood pressure (MBP) were observed between PSTN‐hM3Dq and PSTN‐EGFP mice (Figure 1F,I,J).

In contrast, PSTN‐hM4Di mice exhibited no significant heart rate changes relative to PSTN‐EGFP controls (Figure 1G). However, SBP and DBP were significantly elevated starting 10 min post‐CNO administration, with MBP showing a similar increase (Figure 1H,K,L). Chemogenetic activation of PSTN neurons significantly increased mean pulse pressure within one h, whereas pulse pressure remained unchanged in PSTN‐hM4Di mice (Figure S3A, Supporting Information). Additionally, the rate‐pressure product (RPP), an index of myocardial workload and oxygen consumption,^[^ ^36^ ^]^ was significantly reduced following PSTN neuron activation but increased with inhibition (Figure S3B, Supporting Information).

To further explore the role of PSTN neurons in regulating heart rate and blood pressure under acute hypertensive conditions, norepinephrine (NE, 1 µg g^−1^) was administered subcutaneously alongside CNO (1 mg kg^−1^), with blood pressure recordings initiated 10 min post‐injection. Consistent with previous studies,^[^ ^37^ ^]^ NE induced a blood pressure peak within 5–10 min, returning to baseline after ≈45 min. Under acute hypertensive conditions, PSTN‐hM3Dq mice exhibited reduced heart rate (Figure 1M), attenuated SBP elevation compared to PSTN‐EGFP controls (Figure 1N). No significant differences in DBP or MBP were observed throughout the test period (Figure 1Q,R). Conversely, PSTN‐hM4Di mice showed no significant heart rate changes (Figure 1O) but displayed significantly greater elevations in SBP, DBP, and MBP, with prolonged recovery times to baseline compared to controls (Figure 1P,S,T).

**Figure 2:**
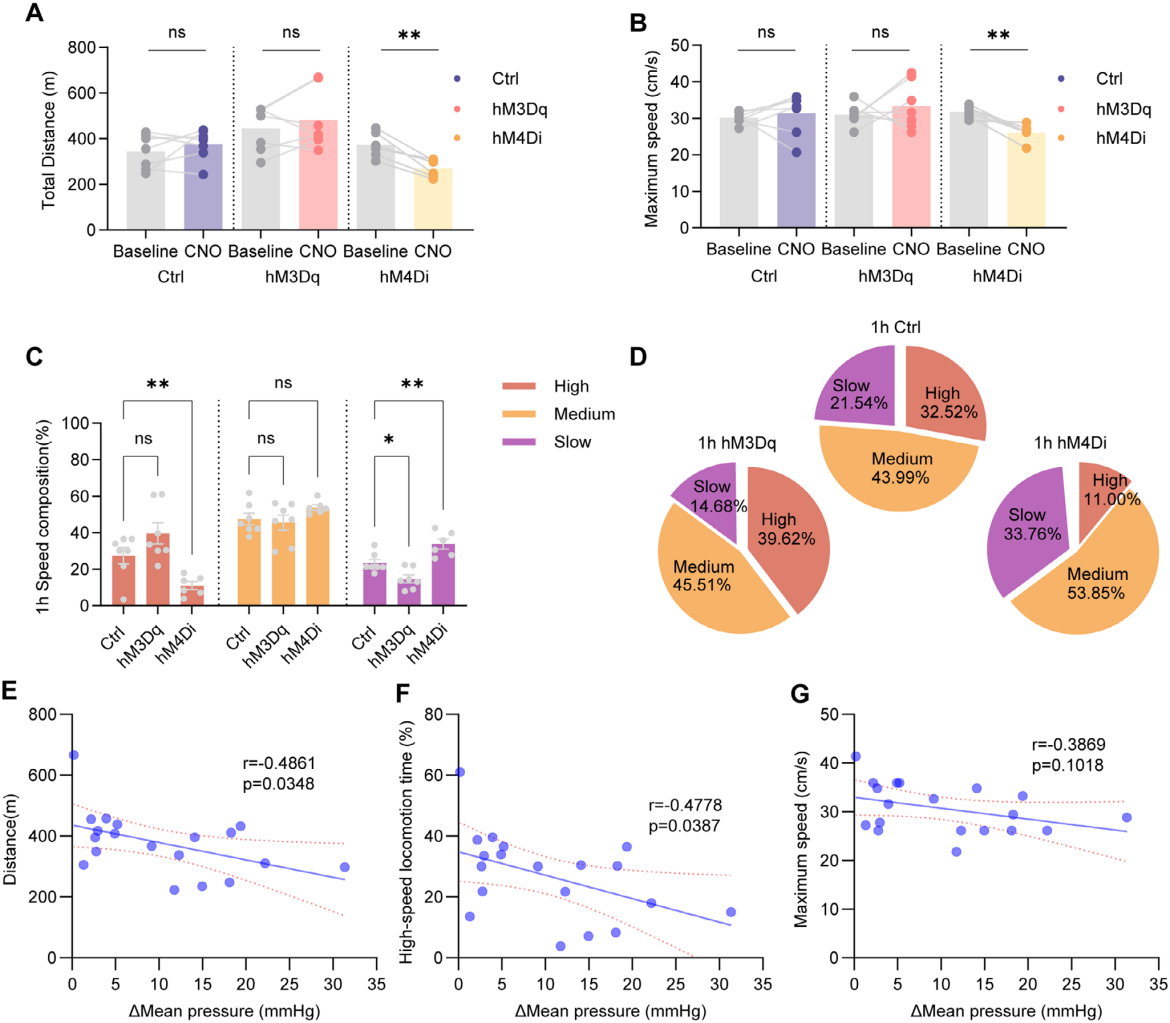
Chemogenetic manipulation of PSTN neurons alters locomotor activity. A,B) Summary of distance and maximum speed in 1‐h wheel running in mice (mCherry mice: n = 7, hM3Dq mice: n = 7; hM4Di mice: n = 6; paired two‐sided t‐test; mCherry vs hM4Di; Total distance: t_5_ = 5.065 *p =* 0.0039; Maximum speed: t_5_ = 4.944, *p*  =   0.0043). C) Summary of 1‐h speed composition in mice (mCherry mice: n = 7, hM3Dq mice: n = 7; hM4Di mice: n = 6; One‐way ANOVA; high‐speed locomotion time: F_2,17 _ = 13.49, *p =*   0.0003; mCherry vs hM4Di, *p =* 0.0030; medium‐speed locomotion time: F_2,17_ = 3.014, *p =* 0.1455; mCherry vs hM4Di, *p =* 0.0616, slow‐speed locomotion time: F_2,17_ = 21.31, *p* < 0.0001; mCherry vs hM3Dq, *p* = 0.0489; mCherry vs hM4Di, *p* = 0.0013). D) Pie charts showing 1‐h speed composition in the mCherry, hM3Dq and hM4Di, excluding immobility data. E) Correlations of total distance and |ΔMBP|, Pearson's *r* = −0.4861, *p* = 0.0348. F) Correlations of time spent in high‐speed locomotion and |ΔMBP|, Pearson's *r* = −0.4778, *p* = 0.0387. G) Correlations of maximum speed and |ΔMBP|, Pearson's *r* = −0.3869, *p* = 0.1018. |ΔMBP|, average amplitude of mean pressure changes. Each circle represents results from one mouse. Data are represented as mean ± SEM (C). ns, *p *> 0.05; *p < 0.05; **p < 0.01. See also Table S1 (Supporting Information).

In the 1‐h running wheel experiment, PSTN‐hM3Dq mice showed no significant differences in total distance or maximum running speed compared to PSTN‐EGFP mice (**Figure** **2**A,B). Conversely, PSTN‐hM4Di mice exhibited significant reductions in both total distance and maximum running speed (Figure 2A,B). Analysis of speed composition revealed notable differences: compared to PSTN‐EGFP mice, PSTN‐hM4Di mice demonstrated a significant reduction in high‐speed locomotion time (PSTN‐hM4Di: 11.00 ± 2.20% vs PSTN‐EGFP: 32.52 ± 2.19%), a significant increase slow‐speed locomotion time (PSTN‐hM4Di: 33.76 ± 2.25% vs PSTN‐EGFP: 21.54 ± 2.19%). Meanwhile, medium‐speed running time increased significantly (PSTN‐hM4Di: 53.85 ± 1.46% vs PSTN‐EGFP: 43.99 ± 2.30%), as did slow‐speed locomotion time (PSTN‐hM4Di: 33.79 ± 2.75% vs PSTN‐EGFP: 21.54 ± 1.14%). In contrast, PSTN‐hM3Dq mice showed no significant changes in high‐speed locomotion time but displayed a significant reduction in slow‐speed locomotion time compared to PSTN‐EGFP mice (PSTN‐hM3Dq:14.68 ± 2.16% vs PSTN‐EGFP: 23.0 ± 1.14%) (Figure 2C,D).

To assess whether PSTN neurons regulate cardiovascular stability and locomotor activity independently or coordinately, we analyzed the relationship between the average amplitude of mean pressure changes (|ΔMBP|) during the chemogenetic manipulation period and locomotor performance in individual mice. We found that |ΔMBP| was negatively correlated with total running distance (Pearson's *r* = −0.4861, *p* = 0.0348) and with time spent in high‐speed locomotion (Pearson's *r* = −0.4778, *p* = 0.0387). In contrast, there was no significant correlation between |ΔMBP| and maximum running speed (Pearson's *r* = ‐0.3869, *p* = 0.1018) (Figure 2E–G).

Collectively, these findings demonstrate that PSTN neurons are essential for maintaining cardiovascular homeostasis, which is critical for supporting voluntary locomotion, particularly during high‐speed, energy‐demanding activities.

### 2.3 Population Activities of PSTN*^Vglut2^* Neurons Increase During Acute Hypertension

**Figure 3:**
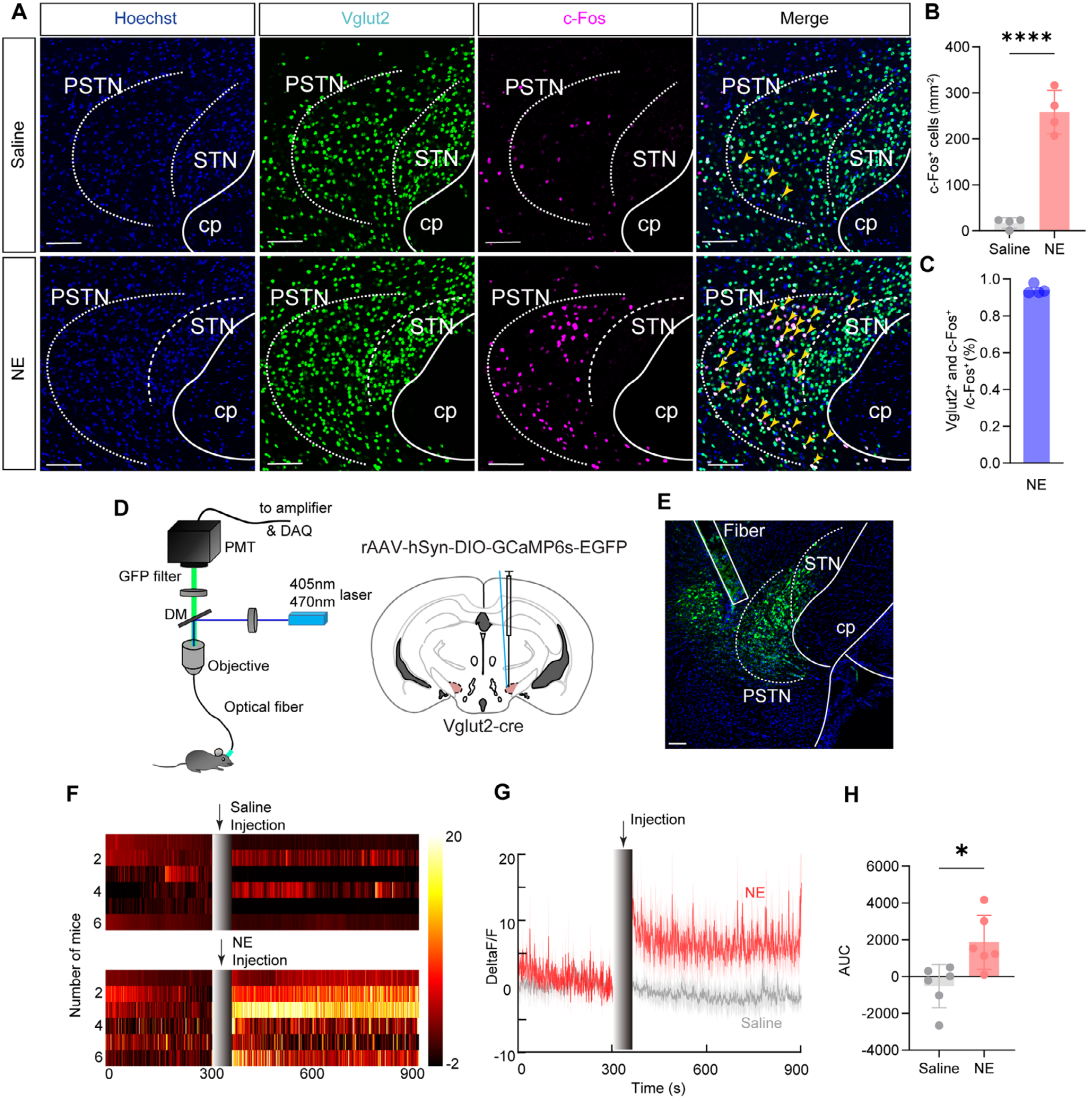
Population activity of PSTN*^Vglut2^* neurons during acute hypertension. A) Representative photomicrographs of the PSTN depicting Hoechst (blue), *Vglut2^+^* neurons (green), c‐Fos^+^ (magenta) and merge (white) images of *Vglut2::H2B* mice after administration of saline or NE. B) Quantitative analysis of c‐Fos^+^ neurons in the PSTN. (Saline n = 4, NE n = 4, unpaired two‐sided t‐test; t_6_ = 9.935, *p*<0.0001) C Percentage of c‐Fos cells expressing *Vglut2* (n = 4). D) Schematic of the in vivo recording configuration. E) Representative image showing GCaMP6s‐EGFP‐labeled PSTN*^Vglut2^* neurons and the position of the tip of the fiber optic in the PSTN (n = 6). The position of the tip of the fiber optic in the PSTN. F) Heatmaps of all recorded trials showing the changes in calcium signals of PSTN*^Vglut2^* neurons before and following vehicle or NE administration. G) The calcium signals of PSTN*^Vglut2^* neurons before and after saline (gray trace) ± SEM (gray shading) or NE (red trace) ± SEM (red shading) administration. Arow heads indicate time of injection. H) AUC of calcium signals following saline/NE injection (n = 6, unpaired two‐sided t‐test; t_10_ = 3.094, *p*<0.0114). PSTN: parasubthalamic nucleus, STN: subthalamic nucleus, cp: cerebral peduncle. Scale bar: 100 µm. See also Table S1 (Supporting Information).

The PSTN is primarily a glutamatergic structure, characterized by abundant expression of the *Slc17a6* gene, encoding *Vglut2*.^[^ ^28^, ^30^ ^]^ To determine whether PSTN*^Vglut2^* neurons respond to acute hypertension, we crossed *Vglut2‐Cre* mice with *H2B‐GFP* reporter mice, generating *Vglut2::H2B* mice, enabling nuclear GFP labeling of *Vglut2*‐positive neurons throughout the brain. We then assessed neuronal activation by performing c‐Fos immunostaining in brain slices from *Vglut2::H2B* mice following intraperitoneal administration of either saline or norepinephrine (NE; 1 µg g^−1^) (**Figure** **3**A). Compared to saline, NE administration increased c‐Fos expression in the PSTN of *Vglut2::H2B* mice (Figure 3B). Immunofluorescence staining revealed that ≈90% of the c‐Fos signal co‐localized with PSTN*^Vglut2^* neurons (Figure 3C).

To investigate real‐time neuronal activity of PSTN*^Vglut2^* neurons during acute hypertension, we delivered an AAV encoding the calcium indicator GCaMP6s (rAAV‐hSyn‐DIO‐GCaMP6s) into the PSTN of *Vglut2‐Cre* mice and implanted an optical fiber above the target region to record calcium signals (Figure 3D,E). To minimize interference from spontaneous behaviors such as walking, sniffing, and rearing, experiments were conducted under anesthesia.^[^ ^38^ ^]^ Calcium signals in PSTN*^Vglut2^* neurons increased rapidly following NE injection and remained elevated during acute hypertension (Figure 3F,G). The area under the curve (AUC) of calcium signals 3–8 min after NE (1 µg g^−1^) injection was significantly greater than that of the saline group (Figure 3H). These findings indicate that PSTN*^Vglut2^* neurons is associated with acute elevated blood pressure.

### 2.4 PSTN*^Vglut2^* Neurons Modulate Cardiovascular Function and Locomotor Behavior

**Figure 4:**
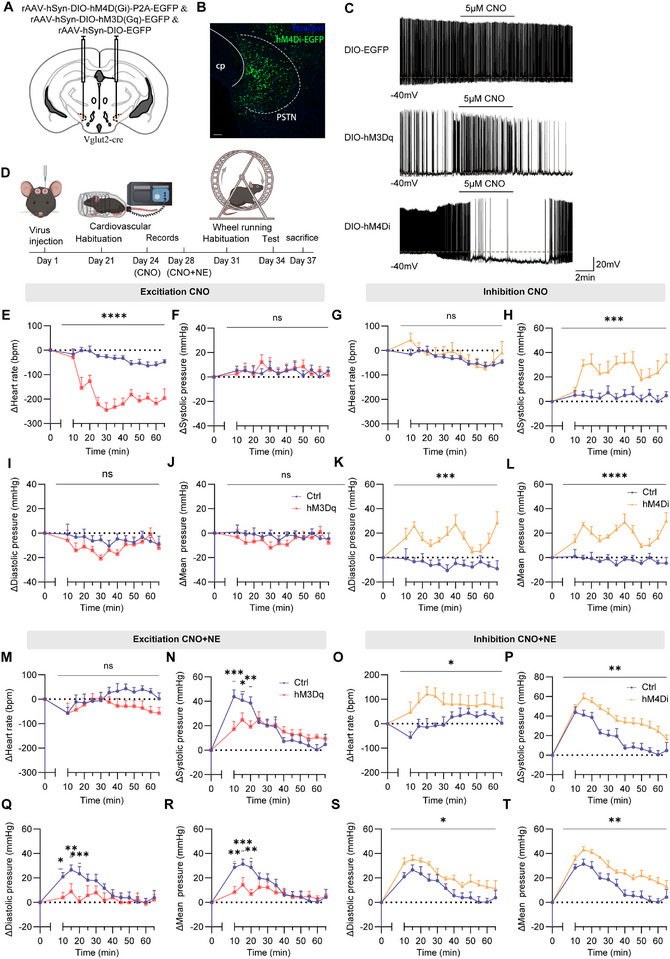
Chemogenetic manipulation of PSTN*^Vglut2^* neurons alters heart rate and blood pressure. A) Schematic of microinjection into the PSTN of the *Vglut2‐Cre* mice. B) Representative photomicrographs of the PSTN depicting EGEP expression from a *Vglut2‐Cre* mice. PSTN: parasubthalamic nucleus. cp: cerebral peduncle. Scale bar: 100 µm. C) Representative voltage traces recorded from an mCherry, hM3Dq or hM4Di ‐expressing during the application of CNO. D) Schematic of the protocol in experiments. E,F,I,J) Summary of changes in heart rate and blood pressure in *Vglut2‐Cre* mice after administration of CNO, EGFP mice: n = 7; hM3Dq mice: n = 6 E) ∆Heart rate (Two‐way ANOVA F_1,11_ = 45.69, *p* < 0.0001). F) ∆Systolic pressure I) ∆Diastolic pressure J) ∆Mean pressure (Two‐way ANOVA; not statistically significant). G,H,K,L) Summary of changes in heart rate and blood pressure in *Vglut2‐Cre* mice after administration of CNO, EGFP mice: n = 6; hM4Di mice: n = 6. G) ∆Heart rate (Two‐way ANOVA; not statistically significant). H) ∆Systolic pressure (Two‐way ANOVA F_1,11_ = 11.24, *p =* 0.0065). K) ∆Diastolic pressure (Two‐way ANOVA; F_1,11_ = 18.42, *p =* 0.0013). L) ∆Mean pressure (Two‐way ANOVA; F_1,11_ = 35.06, *p* < 0.0001). M,N,Q,R) Summary of changes in heart rate and blood pressure in *Vglut2‐Cre* mice after administration of CNO and NE, EGFP mice: n = 6; hM3Dq mice: n = 6. M) ∆Heart rate (Two‐way ANOVA F_1,10_ = 1.883, *p* = 0.2000). N) ∆Systolic pressure (Two‐way ANOVA F_1,10_ = 0.1020, *p* = 0.7560 (10‐65 min); 10 min: *p* = 0.0001; 15 min: *p =* 0.0193; 20 min *p* = 0.0047). Q) ∆Diastolic pressure (Two‐way ANOVA F_1,10_ = 2.278, *p* = 0.1621 (10‐65 min); 10 min: *p* = 0.0123; 15min: *p* = 0.0.0093; 20min *p* = 0.0009). R) ∆Mean pressure (Two‐way ANOVA F_1,10_ = 1.476, *p* = 0.2524 (10‐65 min); 10min: *p* = 0.0010; 15min: *p* = 0.0057; 20min *p* = 0.0004). O,P,S,T) Summary of changes in heart rate and blood pressure in *Vglut2‐Cre* mice after administration of CNO and NE, EGFP mice: n = 6; hM4Di mice: n = 6. O) ∆Heart rate (Two‐way ANOVA; not statistically significant). P) ∆Systolic pressure (Two‐way ANOVA F_1,10_ = 15.33, *p =* 0.0029). S) ∆Diastolic pressure (Two‐way ANOVA F_1,10_ = 8.193, *p =* 0.0169). T) ∆Mean pressure (Two‐way ANOVA F_1,10_ = 13.66, *p* = 0.0041). NE, norepinephrine. Data are represented as mean ± SEM (E‐T). Δ represents the amount of change in cardiovascular parameters relative to baseline. Two‐way repeated measures ANOVA followed by Bonferroni post hoc test; ns, *p *> 0.05; *p < 0.05; **p < 0.01; ***p < 0.001 and ****p < 0.0001. See also Table S1 (Supporting Information).

To investigate the role of PSTN*^Vglut2^* neuron in regulating cardiovascular function and locomotor behavior, we expressed *Cre*‐dependent DREADDs in the PSTN of *Vglut2‐Cre* mice. Specifically, AAV‐DIO‐hSyn‐hM3D(Gq)‐EGFP (PSTN*^Vglut2^*‐hM3Dq), AAV‐hSyn‐DIO‐hM4D(Gi)‐EGFP (PSTN*^Vglut2^*‐hM4Di), or AAV‐hSyn‐DIO‐EGFP (PSTN*^Vglut2^*‐EGFP) was bilaterally injected into the PSTN (**Figure** **4**A). Imaging of coronal sections confirmed precise, *Cre*‐dependent expression in PSTN*^Vglut2^* neurons (Figure 4B). DREADD efficacy was validated using whole‐cell current‐clamp recordings in acute PSTN slices, which showed that CNO (10 µM, 6 min) significantly increased spontaneous action potential frequency in hM3Dq‐expressing PSTN*^Vglut2^* neurons, and decreased in hM4Di‐expressing PSTN*^Vglut2^* neurons (Figure 4C).

Following habituation and testing procedures as described in Section 4.10 (Figure 4D), PSTN*^Vglut2^*‐hM3Dq mice exhibited a significant reduction in heart rate from 10 min post‐CNO injection (Figure 4E). No significant changes in SBP were observed compared to baseline, while DBP and MBP showed non‐significant downward trends (Figure 4F,I,J). Mean pulse pressure was significantly increased and RPP was significantly reduced throughout the testing period following CNO administration (Figure S4A,B, Supporting Information). Conversely, PSTN*^Vglut2^*‐hM4Di mice exhibited a significant increase in SBP beginning 15 min post‐CNO, persisting with greater variability throughout the testing period (Figure 4H). DBP and MBP were significantly elevated from 10 min onward, remaining elevated throughout the recording period (Figure 4K,L). Mean pulse pressure showed no significant change compared to baseline in PSTN*^Vglut2^*‐EGFP mice, whereas RPP was significantly elevated (Figure S4A,B, Supporting Information).

To further investigate whether PSTN neurons play a role in regulating heart rate and blood pressure under acute hypertensive conditions, we utilized NE (1 µg g^−1^) to induce acute hypertension. Under these conditions, PSTN*^Vglut2^*‐hM3Dq mice exhibited no significant effect on heart rate compared to PSTN*^Vglut2^*‐EGFP mice (Figure 4M). However, they exhibited attenuated increases in SBP, DBP, and MBP during the rapid blood pressure rise, with no significant difference in the time required for these parameters to return to baseline (Figure 4N,Q,R). In contrast, PSTN*^Vglut2^*‐hM4Di mice exhibited significant increases in heart rate and blood pressure compared to PSTN*^Vglut2^*‐EGFP mice. Specifically, heart rate peaked ≈20 min after CNO injection, despite a slight decline thereafter, and remained significantly higher than in PSTN*^Vglut2^*‐EGFP controls throughout the experiment (Figure 4O). In PSTN*^Vglut2^*‐hM4Di mice, SBP, DBP, and MBP elevations were more pronounced, with significantly prolonged recovery times to baseline (Figure 4P,S,T).

**Figure 5:**
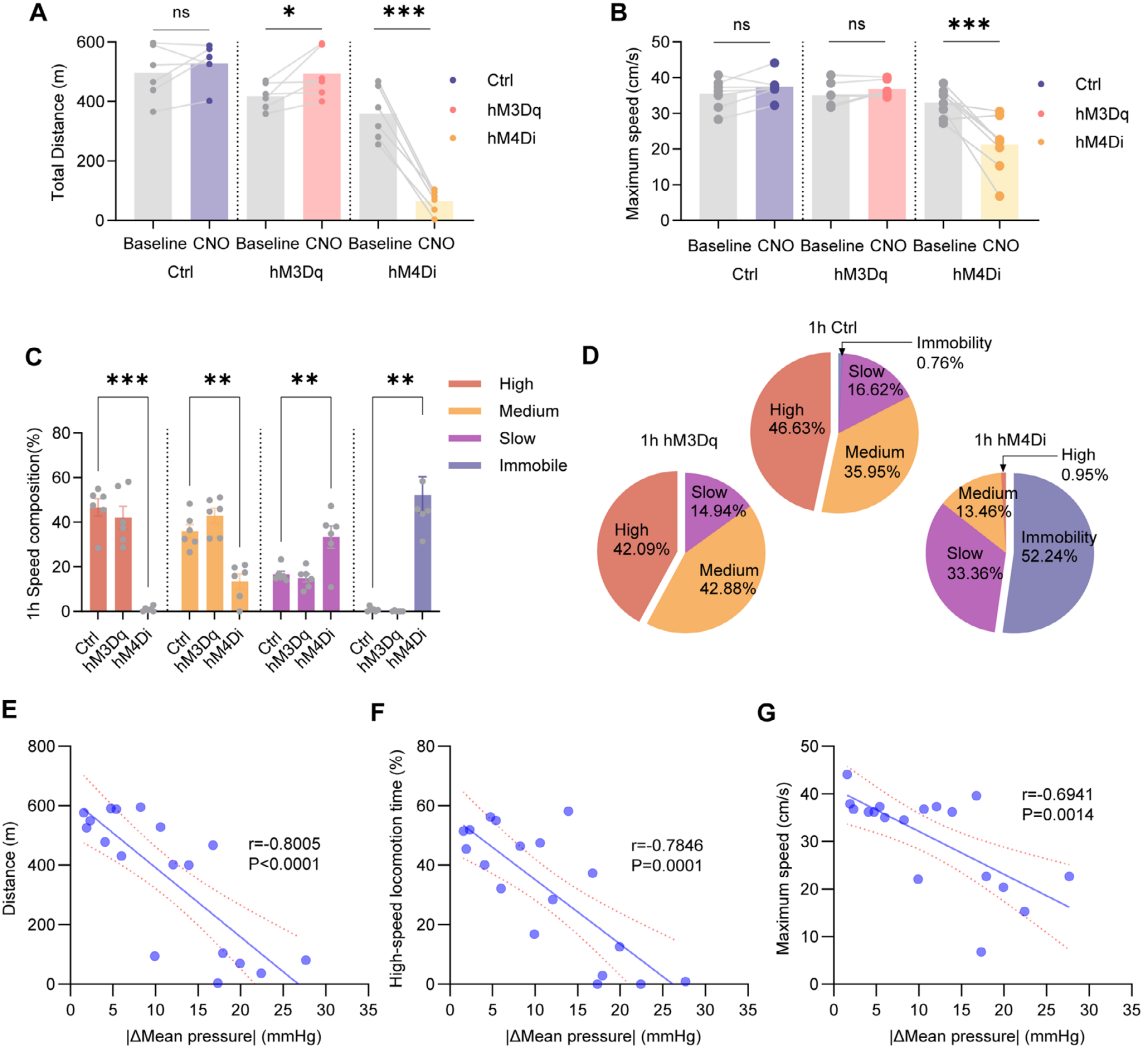
Chemogenetic manipulation of PSTN*^Vglut2^* neurons alters locomotor activity. A,B) Summary of distance and maximum speed in 1‐h wheel running in mice (EGFP mice: n = 6, hM3Dq mice: n = 6; hM4Di mice: n = 6; paired two‐sided t‐test; Total distance: EGFP mice: n = 6, not statistically significant; hM3Dq mice: n = 6, t_5_ = 2.637, *p* = 0.0461; hM4Di mice: n = 6; t_5_ = 10.13, *p* = 0.0002; Maximum speed: EGFP mice: n = 6, not statistically significant; hM3Dq mice: n = 6, not statistically significant; hM4Di mice: n = 6; t_5_ = 8.298, *p* = 0.0004; C) Summary of 1‐h speed composition in mice (EGFP mice: n = 6, hM3Dq mice: n = 6; hM4Di mice: n = 6; One‐way ANOVA; high‐speed locomotion time: F_2,15 _ = 46.50, *p* < 0.0001 ; EGFP vs hM4Di, *p* < 0.0001; medium‐speed locomotion time: F_2,15_ = 22.02, *p* <0.0001; EGFP vs hM4Di, *p* = 0.0004; slow‐speed locomotion time: F_2,17_ = 10.18, *p* = 0.0016; EGFP vs hM4Di, *p* = 0.004; immobility time: F_2,15_ = 40.46, *p* < 0.0001). D) Pie charts showing 1‐h speed composition in EGFP, hM3Dq, and hM4Di mice, with immobility data excluded for hM3Dq mice. E) Correlations of total distance and |ΔMBP|, Pearson's *r* =−0.8005, *p* < 0.0001. F) Correlations of time spent in high‐speed locomotion and |ΔMBP|, Pearson's *r* = −0.7846, *p* = 0.0001. G) Correlations of maximum running speed and |ΔMBP|, Pearson's *r* = −0.6941, *p* = 0.0014. |ΔMBP|, average amplitude of mean pressure changes. Each circle represents results from one mouse. Data are represented as mean ± SEM (C). ns, *p* > 0.05; *p < 0.05; **p < 0.01 and ***p < 0.001. See also Table S1 (Supporting Information).

In a 1‐h running wheel experiment, intraperitoneal CNO injection in PSTN*^Vglut2^*‐hM3Dq mice showed a significant increase in total distance, while no significant effect on maximum speed compared to baseline (**Figure** **5**A,B). PSTN*^Vglut2^*‐hM4Di mice exhibited significant reductions in both total distance and maximum speed (Figure 5A,B). Analysis of speed composition revealed statistically significant difference: compared to PSTN*^Vglut2^*‐EGFP mice, PSTN*^Vglut2^*‐hM4Di mice demonstrated a significant reduction in high‐speed locomotion time (PSTN*^Vglut2^*‐hM4Di: 0.95 ± 0.45% vs PSTN*^Vglut2^*‐EGFP: 46.64 ± 3.89%), medium‐speed locomotion time (PSTN*^Vglut2^*‐hM4Di: 13.46 ± 3.36% vs PSTN*^Vglut2^*‐EGFP: 35.95 ± 3.13%), while slow‐speed locomotion time (PSTN*^Vglut2^*‐hM4Di:33.36 ± 5.03% vs PSTN*^Vglut2^*‐EGFP: 16.62 ± 1.38%), and immobility time significantly increased (PSTN*^Vglut2^*‐hM4Di: 52.24 ± 8.13% vs PSTN*^Vglut2^*‐EGFP:0.79 ± 0.39%). PSTN*^Vglut2^*‐hM3Dq mice showed no significant differences in speed composition compared to PSTN*^Vglut2^*‐EGFP mice (Figure 5C,D).

To evaluate the relationship between cardiovascular stability and locomotor performance, we correlated the absolute amplitude of mean blood pressure fluctuations during chemogenetic manipulation (|ΔMBP|) with three locomotor metrics. |ΔMBP| showed strong negative correlations with total distance (Pearson's *r* = −0.8005, *p* < 0.0001), duration of high‐speed locomotion (Pearson's *r* = −0.7846, *p* = 0.0001), and peak running speed (Pearson's *r* = −0.6941, *p* = 0.0014) (Figure 5E–G).

These findings demonstrate that PSTN*^Vglut2^* neurons mitigates blood pressure elevations during acute hypertensive challenges without impairing locomotor activity. In contrast, inhibition of PSTN*^Vglut2^* neurons exacerbates hypertensive responses, delays recovery, and significantly reduces both overall locomotor activity and maximum running speed, effects strongly correlated with impaired blood pressure homeostasis. The more pronounced cardiovascular and locomotor changes observed with PSTN*^Vglut2^* neuron manipulation, compared to non‐specific PSTN neuron manipulation, likely result from circuit‐specific mechanisms and antagonistic interactions among other PSTN neuronal subpopulations.

### 2.5 Downstream Targets of PSTN*^Vglut2^* Neurons and Their Synaptic Connections with the NTS

**Figure 6:**
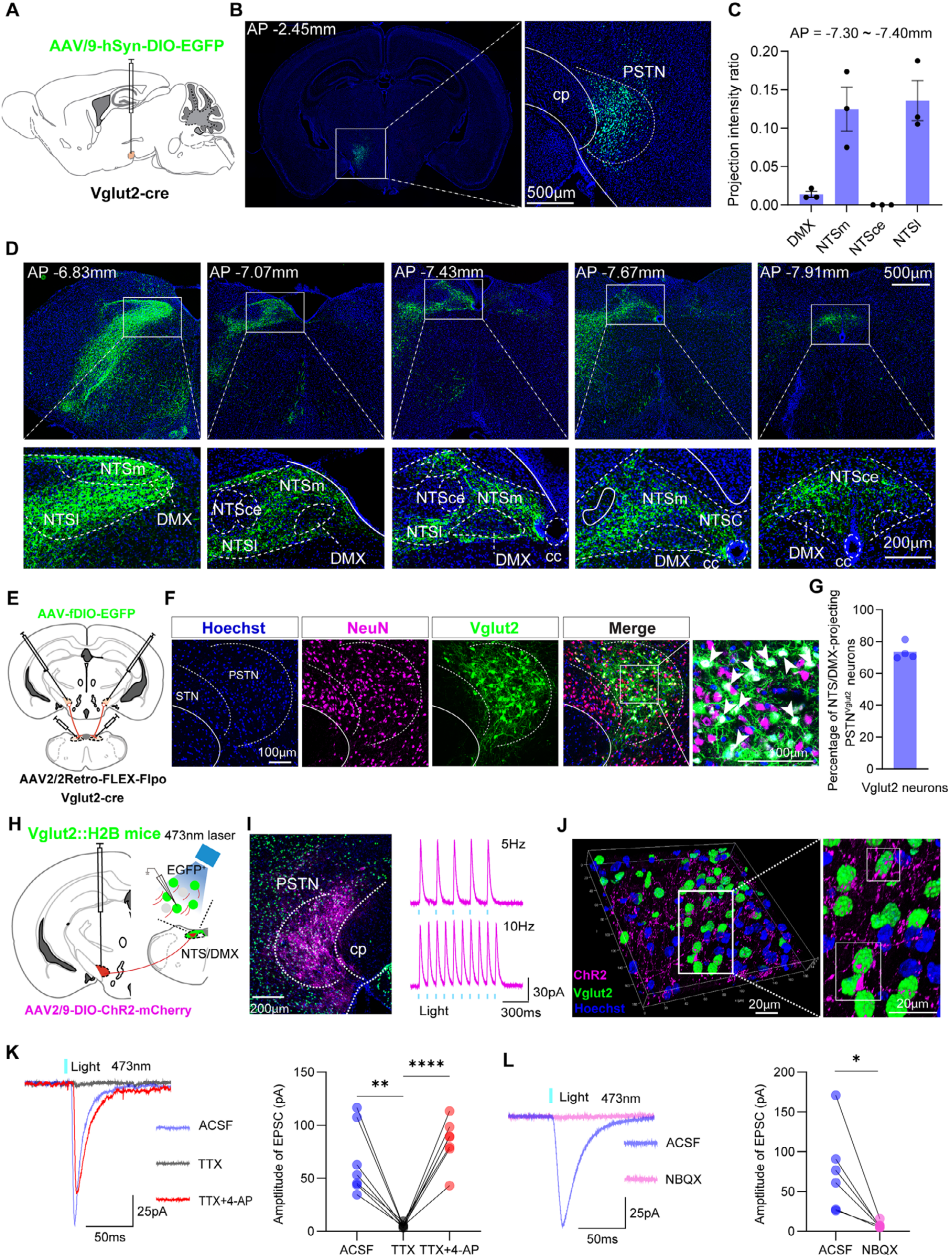
Identification of PSTN‐NTS monosynaptic glutamatergic pathway. A) Schematic of unilateral AAV2/9‐hSyn‐DIO‐EGFP injection into the PSTN of the *Vglut2‐Cre* mice. B) Representative image of virus injection site in the PSTN, EGFP‐labeled. Scale bar: 500 µm. C) Quantitative analysis of projection intensity NTS and DMX (AP = −7.3 ≈ −7.4mm), n = 3. D) representative images of PSTN*^Vglut2^* neurons projecting to NTS subregions (EGFP‐labeled) and Scale bar: 200 µm. E) Schematic of bilateral AAV‐ fDIO‐ EGFP injection into the PSTN and bilateral RetroAAV‐ FLEX‐ Flpo injection into the NTS of the *Vglut2‐Cre* mice. F) Representative photomicrographs of the PSTN depicting Hoechst (blue), NeuN (magenta), *Vglut2^+^* neurons (green) and merge (white) images of *Vglut2‐Cre* mice. Scale bar: 100 µm. G) Statistical analysis of the percentage of PSTN*^Vglut2^* neurons projecting to NTS, n = 3. H) Schematic of virus injection into the PSTN of the *Vglut2::H2B* mice and optical activation of PSTN*^Vglut2^* terminals with simultaneous whole‐cell patch‐clamp recordings of EGFP^+^ NTS*^Vglut2^* neurons. I) Representative image of virus injection site in the PSTN (mCherry‐labeled) and sample traces of action potentials evoked by light (473 nm, 5 ms, blue line) recorded from mCherry^+^ PSTN*^Vglut2^* neurons in acute brain slices. J) Representative images of the axon projection targets of mCherry^+^ PSTN*^Vglut2^* neurons of *Vglut2::H2B* mice, Scale bar: 20 µm. K) Representative traces (left) and summarized data (right) for light‐evoked postsynaptic currents (EPSCs) recorded from NTS^Vglut2^ neurons (paired two‐sided t‐test, n = 7 neurons from 3 mice. ACSF vs TTX, t_6_ = 4.892, *p* = 0.0027; TTX vs TTX+4‐AP, t_6_ = 3.889, *p* < 0.0001). L) Representative traces (left) and summarized data (right) for light‐evoked postsynaptic currents (EPSCs) recorded from NTS^*Vglut2*^ neurons (paired two‐sided t‐test, n = 7 neurons from 3 mice. ACSF vs NBQX, t_6_ = 4.892, *p* = 0.0208). Each circle represents results from one mouse. *p < 0.05; **p < 0.01 and ****p < 0.0001. See also Table S1 (Supporting Information).

Since the PSTN lacks direct neural connections with peripheral systems, it likely regulates cardiovascular homeostasis and locomotion via downstream brain regions. To identify the downstream targets of PSTN*^Vglut2^* neurons that regulate cardiovascular responses and locomotion, we injected AAV2/9‐DIO‐EGFP into the PSTN of *Vglut2‐Cre* mice (**Figure** **6**A,B). Using anterograde fluorescence tracing, we systematically surveyed 64 brain regions. In these mice, EGFP‐labeled axon terminals originating from the PSTN were detected throughout the brain, including the cortex, basal forebrain, basal ganglia, amygdaloid complex, bed nucleus of the stria terminalis, thalamus, hypothalamus, midbrain, pons, and medulla (Figure S5A, Supporting Information).

The PSTN*^Vglut2^* neurons exhibited robust connectivity with limbic system‐associated regions, such as the visceral area, medial and lateral subdivisions of the bed nucleus of the stria terminalis (Figure S5B, Supporting Information), and the central amygdaloid nucleus–‐particularly its medial, capsular, and lateral subdivisions, with a predominance in the medial subdivision (Figure S5C, Supporting Information). In the thalamus, PSTN*^Vglut2^* neurons prominently targeted the lateral habenula, paraventricular nucleus, and reuniens nucleus (Figure S5D, Supporting Information). Additionally, dense projections were observed in the midbrain, particularly to regions associated with locomotion and motor control, including the ventral tegmental area, lateral periaqueductal gray, and substantia nigra reticulata (Figure S5E, Supporting Information).

The medulla serves as the central integrative center for cardiovascular control. It processes signals from peripheral receptors, mediates autonomic reflexes (sympathetic and parasympathetic), directly governs heart rate and vascular tone, and coordinates with higher brain centers to maintain blood pressure and heart rate homeostasis.^[^ ^6^ ^]^ Within the medulla, PSTN*^Vglut2^* neurons predominantly targeted the NTS, innervating both medial and lateral subdivisions across its full rostrocaudal extent. Although projections were also observed in the rostral DMX, these were markedly less dense than those in the NTS (Figure 6C,D).

To quantify these projections, we employed an intersectional viral strategy: AAV2/2Retro‐FLEX‐Flpo was bilaterally injected into the NTS, and AAV2/9‐fDIO‐EGFP into the PSTN of *Vglut2‐Cre* mice. This approach selectively labeled NTS‐projecting PSTN*^Vglut2^* neurons. Immunohistochemical staining for NeuN revealed clear mCherry expression in the PSTN, and fluorescence colocalization analysis indicated that 73.53 ± 2.6% of NeuN⁺/mCherry⁺ neurons were also EGFP⁺ (Figure 6E−G).

To determine the functional synaptic transmission between PSTN*^Vglut2^* and NTS*^Vglut2^* neurons, we injected AAV2/9‐DIO‐ChR2‐mCherry into the PSTN of *Vglut2::H2B* mice, achieving selective ChR2‐mCherry expression in PSTN*^Vglut2^* neurons. Whole‐cell patch‐clamp recordings were performed on acute brainstem slices to measure light‐evoked excitatory postsynaptic currents (EPSCs) in GFP‐labeled NTS*^Vglut2^* neurons (Figure 6H). We first verified ChR2 functionality in PSTN*^Vglut2^* neurons by confirming reliable blue light‐induced currents at 5 Hz and 10 Hz in brain slices (Figure 6I). Subsequent photostimulation of ChR2‐expressing PSTN*^Vglut2^* axon terminals, at a holding potential of −70 mV, reliably evoked EPSCs in NTS*^Vglut2^* neurons. These light‐evoked EPSCs were eliminated by tetrodotoxin (TTX) but persisted in the presence of TTX and 4‐aminopyridine (4‐AP) (Figure 6K). Furthermore, application of the AMPA receptor antagonist 2,3‐dihydroxy‐6‐nitro‐7‐sulfamoyl‐benzo[f] quinoxaline (NBQX) significantly attenuated these EPSCs (Figure 6L). Collectively, these results demonstrate that PSTN*^Vglut2^* neurons form direct, functional glutamatergic synapses onto NTS*^Vglut2^* neurons.

The NTS serves as a key integrator of baroreceptor input, modulating parasympathetic output through projections to the Amb and DMX.^[^ ^39^ ^]^ Our finding that most PSTN*^Vglut2^* neurons project to the NTS suggests a specialized autonomic circuit. Although PSTN→NTS projections have been previously reported,^[^ ^35^ ^]^ their physiological role in cardiovascular regulation remains unexplored. Therefore, we hypothesize that PSTN*^Vglut2^* neurons control heart rate, blood pressure, and autonomic aspects of locomotion through parasympathetic output via the NTS.

### 2.6 NTS‐projecting PSTN*^Vglut2^* Neurons Modulate Cardiovascular Function and Locomotor Behavior

**Figure 7:**
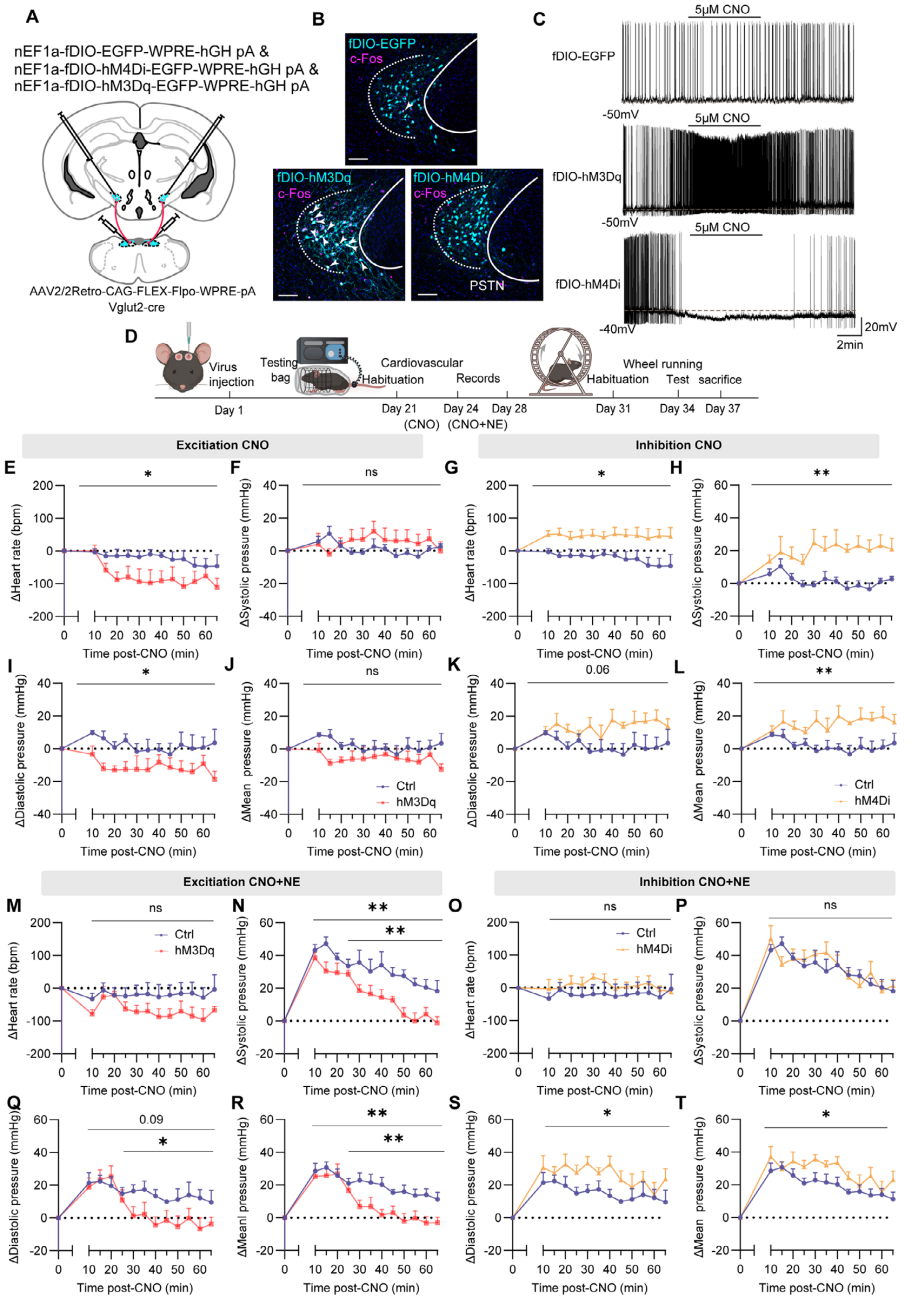
Chemogenetic manipulation of NTS‐projecting PSTN*^Vglut2^* neurons alters heart rate and blood pressure. A) Schematic of microinjection into the PSTN of the *Vglut2‐Cre* mice. B) Representative photomicrographs of the PSTN depicting hoechst (blue), EGFP (cyan), c‐Fos+ (Magenta), and merge (white) images of PSTN‐EGFP, PSTN‐hM3Dq and PSTN‐hM4Di mice after administration of CNO. White arrows point to merged cell bodies. PSTN: parasubthalamic nucleus. Scale bar: 100 µm. C) Representative voltage traces recorded from an mCherry, hM3Dq or hM4Di ‐expressing during the application of CNO. D) Schematic of the protocol in experiments. E,F,I,J) Summary of changes in heart rate and blood pressure in *Vglut2‐Cre* mice after administration of CNO, EGFP mice: n = 6; hM3Dq mice: n = 6. E) ∆Heart rate (Two‐way ANOVA F_1,10_ = 5.763, *p* = 0.0373). F) ∆Systolic pressure (Two‐way ANOVA; not statistically significant) I) ∆Diastolic pressure (Two‐way ANOVA F1,10 = 5.134, *p* = 0.0469) J) ∆Mean pressure (Two‐way ANOVA; not statistically significant). G,H,K,L) Summary of changes in heart rate and blood pressure in *Vglut2‐Cre* mice after administration of CNO, EGFP mice: n = 6; hM4Di mice: n = 6. G) ∆Heart rate (Two‐way ANOVA; F_1,10_ = 7.931, *p* = 0.0183). H) ∆Systolic pressure (Two‐way ANOVA; F_1,10_ = 13.32, *p* = 0.0045). K) ∆Diastolic pressure (Two‐way ANOVA; F_1,10_ = 18.42, *p* = 0.0633). L) ∆Mean pressure (Two‐way ANOVA; F_1,10_ = 9.706, *p* = 0.0110). M,N,Q,R) Summary of changes in heart rate and blood pressure in *Vglut2‐Cre* mice after administration of CNO and NE, EGFP mice: n = 6; hM3Dq mice: n = 6. M) ∆Heart rate (Two‐way ANOVA F_1,10_ = 2.548, *p* = 0.1415). N) ∆Systolic pressure (Two‐way ANOVA F_1,10_ = 13.69, *p* = 0.0041. Q) ∆Diastolic pressure (Two‐way ANOVA, 30–65min, F_1,10_ = 5.188, *p* = 0.0460). R) ∆Mean pressure (Two‐way ANOVA, 30–65min, F_1,10_ = 12.41, *p* = 0.0055). O,P,S,T) Summary of changes in heart rate and blood pressure in *Vglut2‐Cre* mice after administration of CNO and NE, EGFP mice: n = 6; hM4Di mice: n = 6. O) ∆Heart rate (Two‐way ANOVA; not statistically significant). P) ∆Systolic pressure (Two‐way ANOVA; not statistically significant). O) ∆Diastolic pressure (Two‐way ANOVA; F_1,10_ = 5.093, *p =* 0.0476). T) ∆Mean pressure (Two‐way ANOVA; F_1,10_ = 5.835, *p =* 0.0363). NE, norepinephrine. Data are represented as mean ± SEM (E‐T). Δ represents the amount of change in cardiovascular parameters relative to baseline. Two‐way repeated measures ANOVA followed by Bonferroni post hoc test; ns, *p *> 0.05; *p < 0.05 and **p < 0.01. See also Table S1 (Supporting Information).

To investigate the role of NTS‐projecting PSTN*^Vglut2^* neurons in regulating cardiovascular and locomotor functions, we employed a dual‐recombinase chemogenetic approach. *Vglut2*‐*Cre* mice received simultaneous injections of retroAAV‐FLEX‐FlpO into the NTS and AAV‐fDIO‐hM3Dq‐EGFP, AAV‐fDIO‐hM4Di‐EGFP, or AAV‐fDIO‐EGFP into the PSTN (**Figure** **7**A), yielding EGFP‐tagged DREADDs exclusively in NTS‐projecting PSTN*^Vglut2^* neurons. Immunohistochemical analysis revealed robust co‐labeling of c‐Fos with hM3Dq‐expressing NTS‐projecting PSTN*^Vglut2^* neurons following CNO administration (1 mg kg^−1^, i.p.), whereas no co‐labeling of c‐Fos was detected in hM4Di‐expressing NTS‐projecting PSTN*^Vglut2^* neurons (Figure 7B). Whole‐cell current‐clamp recordings confirmed that bath application of CNO (10 µM, 6 min) significantly increased spontaneous action potential frequency in hM3Dq‐expressing neurons and suppressed in hM4Di‐expressing neurons (Figure 7C). Together, these data validate our approach for bidirectional chemogenetic manipulation of NTS‐projecting PSTN*^Vglut2^* neurons both in vivo and in vitro.

Following habituation and testing procedures as described in Section 4.10 (Figure 7C) Chemogenetic activation of NTS‐projecting PSTN*^Vglut2^* neurons produced a sustained reduction in heart rate beginning at 15 min post‐CNO injection and persisting throughout the recording period (Figure 7E). DBP likewise decreased significantly compared to controls from 15 min onward, while SBP and MBP showed no significant changes (Figure 7F,I,J). In contrast, chemogenetic inhibition of NTS‐projecting PSTN*^Vglut2^* neurons significantly elevated heart rate at 10 min (Figure 7G) and elevated SBP and MBP from 15 min post‐CNO inection, both of which remained significantly higher than controls for the duration of the test (Figure 7H,L). DBP tended to rise following chemogenetic NTS‐projecting PSTN*^Vglut2^* neurons inhibition, but this increase did not reach statistical significance (Figure 7K). Moreover, activation increased mean average pulse pressure over the test period without altering RPP, whereas inhibition significantly elevated RPP without affecting pulse pressure (Figure S6A,B, Supporting Information).

To further explore the role of NTS‐projecting PSTN*^Vglut2^* neurons in regulating heart rate and blood pressure under acute hypertensive conditions, mice received subcutaneous NE (1 µg g^−1^) concomitant with CNO administration, and blood‐pressure measurements began 10 min post injection. Under these acute hypertensive conditions, chemogenetic activation or inhibition of NTS‐projecting PSTN*^Vglut2^* neurons did not significantly affect heart rate under acute hypertensive conditions (Figure 7M). However, activating NTS‐projecting PSTN*^Vglut2^* neurons significantly accelerated the recovery of SBP, DBP, and MBP toward baseline, with the most pronounced effect at 30 min post‐CNO (Figure 7N,Q,R). In contrast, inhibition of the NTS‐projecting PSTN*^Vglut2^* neurons prolonged elevations in DBP and MAP, delaying their return to baseline relative to NE controls (Figure 7S,T), while SBP recovery remained statistically unchanged (Figure 7P). These results underscore the critical role of NTS‐projecting PSTN*^Vglut2^* neurons in both maintaining basal blood pressure and promoting rapid normalization following an acute hypertensive challenge.

**Figure 8:**
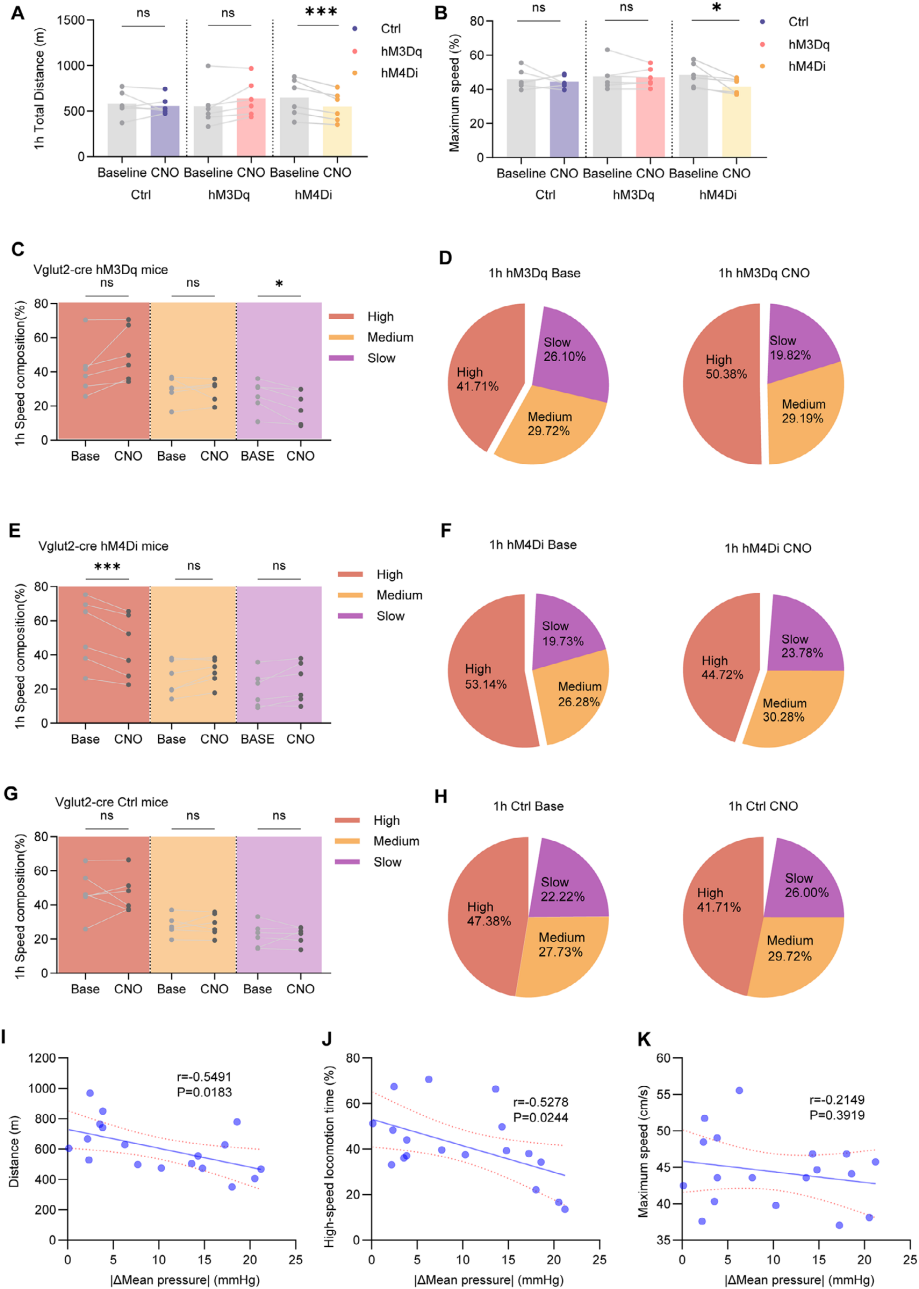
Chemogenetic manipulation of NTS‐projecting PSTN*^Vglut2^* neurons alters locomotor activity. A,B) Summary of distance and maximum speed in 1‐h wheel running in mice (EGFP mice: n = 6, hM3Dq mice: n = 6; hM4Di mice: n = 6; paired two‐sided t‐test; Total distance: EGFP mice: n = 6, not statistically significant; hM3Dq mice: n = 6, t_5_ = 2.251, *p* = 0.0742; hM4Di mice: n = 6; t_5_ = 3.978, *p* = 0.0106; Maximum speed: EGFP mice: n = 6, hM3Dq mice: n = 6, not statistically significant; hM4Di mice: n = 6; t_5_ = 2.667, *p* = 0.0445); C) Summary of 1‐h speed composition in activation group (hM3Dq) mice (n = 6; paired two‐sided t‐test; high‐speed locomotion time: t_5_ = 2.326, *p* = 0.0675; medium‐speed locomotion time: not statistically significant; slow‐speed locomotion time: t_5_ = 3.653, *p* = 0.0147). D,F,H Pie charts showing 1‐h speed composition baseline and after CNO administration in hM3Dq, hM4Di and EGFP mice. E) Summary of 1‐h speed composition in inhibition group (hM4Di) mice (n = 6; paired two‐sided t‐test; high‐speed locomotion time: t_5_ = 7.290, *p* = 0.0008; medium‐speed locomotion time and slow‐speed locomotion time: not statistically significant). G) Summary of 1‐h speed composition in control (EGFP) mice (n = 6; paired two‐sided t‐test; not statistically significant). I) Correlations of total distance and |ΔMBP|, Pearson's *r* = −0.5491, *p* = 0.0183. J) Correlations of time spent in high‐speed locomotion and |ΔMBP|, Pearson's *r* = −0.5278, *p* = 0.0244. K) Correlations of maximum running speed and |ΔMBP|, Pearson's *r* = −0.2149, *p* = 0.3919; |ΔMBP|, average amplitude of mean pressure changes. Each circle represents results from one mouse. Data are represented as mean ± SEM (A,B,C). ns, *p* > 0.05; * *p* < 0.05 and ** *p* < 0.01. See also Table S1 (Supporting Information).

In the 1‐h running wheel experiment, chemogenetic activation of NTS‐projecting PSTN*^Vglut2^* neurons showed no statistically differences in total distance (a trend increase) or maximum running speed compared to the control group (**Figure** 8A,B). Conversely, chemogenetic inhibition of these neurons resulted in significant reductions in both total distance and maximum speed (Figure 8A,B). Analysis of speed composition revealed distinct patterns: in the activation group, slow‐speed locomotion time was significantly reduced (baseline: 26.10 ± 3.66% vs CNO: 19.82 ± 3.93%). Although high‐speed locomotion time showed an increasing trend, the difference was not statistically significant (baseline: 40.71% ± 6.33% vs CNO: 50.38% ± 6.34%) (Figure 8C,D). In contrast, the inhibition group displayed a significant reduction in high‐speed locomotion time (baseline: 53.14% ± 8.02% vs CNO: 44.72% ± 7.48%) and medium‐speed locomotion time (baseline: 26.28 ± 4.08% vs CNO: 30.28 ± 3.10%), while slow‐speed locomotion time showed no statistically significant change (baseline: 19.73 ± 4.26% vs CNO: 23.78 ± 4.82%) (Figure 8E,F). Control group mice showed no no significant changes post‐CNO compared to baseline (Figure 8G,H).

To further assess whether NTS‐projecting PSTN*^Vglut2^* neurons coordinately regulate cardiovascular homeostasis and locomotor functions, we correlated (|ΔMBP|) with locomotor performance. |ΔMBP| was negatively correlated with total running distance (Pearson's *r* = −0.5491, *p* = 0.0183) and high‐speed locomotion time (Pearson's *r* = −0.5278, *p* = 0.0244). In contrast, no significant correlation was observed between |ΔMBP| and maximum running speed (Pearson's *r* = −0.2149, *p* = 0.3919) (Figure 8I–K). Collectively, these findings indicate that NTS‐projecting PSTN*^Vglut2^* neurons contribute to maintaining blood pressure homeostasis while simultaneously coordinating locomotor activity.

## 3 Discussion

The PSTN comprises a discrete cluster of glutamatergic pre‐autonomic neurons within the medial subthalamic region,^[^ ^40^ ^]^ functioning as a critical interoceptive hub that integrates autonomic and homeostatic signals to regulate diverse processes including arousal, exploratory behavior, thermoregulation, and avoidance. These processes fundamentally depend on stable cardiovascular function. Our study identifies a specialized subpopulation of glutamatergic PSTN neurons that are responsive to cardiovascular neural inputs, maintain cardiovascular homeostasis through participation in baroreflex circuits via descending NTS pathways, and coordinately regulate locomotion.

While bidirectional artery‐brain and heart‐brain communication via vagal pathways is well‐established.^[^ ^8^, ^41^ ^]^ Evidence for PSTN‐cardiovascular connectivity has been limited.^[^ ^42^ ^]^ Anatomical evidence from retrograde pseudorabies virus (PRV) tracing in the ventricular myocardium implicates the PSTN as a component of the central cardiac control network.^[^ ^25^ ^]^ and functional studies demonstrate that PSTN stimulation reliably reduces blood pressure and heart rate.^[^ ^33^, ^34^ ^]^ These findings preliminarily establish the PSTN as a key node in cardiovascular modulation and potential central command nucleus for cardiovascular control.

Mechanistically, baroreceptor activation during blood pressure elevation triggers afferent signaling to the central nervous system,^[^ ^43^ ^]^ with vagal activity increasing proportionally to pressure changes.^[^ ^44^ ^]^ Our c‐Fos staining and calcium signaling recordings demonstrate that the activity of PSTN*^Vglut2^* neurons increases during acute hypertension. This suggests that PSTN*^Vglut2^* neurons may receive baroreceptive input, enabling them to exert descending modulation of vagal output. High blood pressure levels acutely evoke a constant tonic firing of baroreceptors, and afferent pathways originating in the NTS relay baroreceptor afferent signals to the broad brain regions involved in integrating autonomic, sensory, interoceptive, and motor input‐output responses to physiological demands to maintain homeostatic balance, thus enabling complex higher‐level brain regions processing to maintain homeostasis.^[^ ^39^ ^]^ The activation of PSTN*^Vglut2^* neurons during elevated blood pressure may result from ascending inputs from the NTS.^[^ ^45^ ^]^ In turn, these inputs also trigger brainstem‐mediated baroreflexes that stabilize arterial pressure within a homeostatic range through beat‐to‐beat adjustments of sympathetic and parasympathetic output to the heart and peripheral vasculature. When arterial pressure rises, parasympathetic (vagal) activity increases.^[^ ^8^, ^39^ ^]^

Chemogenetic manipulations further confirmed that activation of PSTN*^Vglut2^* neurons in awake mice induces a rapid and significant reduction in heart rate under both physiological and hypertensive conditions. Notably, while the hypotensive effect is not significant under physiological conditions, it becomes markedly enhanced during acute hypertension. Activation of PSTN*^Vglut2^* neurons markedly suppresses the amplitude of SBP, DBP, and MBP elevations, reflecting a “damping effect” that likely functions as a protective mechanism to stabilize cardiovascular dynamics. This pattern suggests that PSTN neurons are preferentially recruited under pathological conditions to preserve cardiovascular homeostasis.

In contrast, ablation or inhibition of these neurons disrupts cardiovascular stability, resulting in sustained hypertension under both normal and hypertensive conditions. This functional profile may reflect the contribution of the intrinsic activity of PSTN neurons to baseline cardiovascular regulation and locomotor control. This interpretation is supported by our patch‐clamp recordings, which revealed high spontaneous firing rates in PSTN neurons, indicating their tonic activity under resting conditions. In rest, vagal nerve activity exhibits tonic discharge, and parasympathetic (vagal) influence on the sinoatrial node predominates over sympathetic input.^[^ ^46^ ^]^ These observations suggest that PSTN neurons may act as sensors of blood pressure elevation and participate in cardiovascular regulation via a negative feedback mechanism that enhances parasympathetic output. Such a system is essential for maintaining physiological stability amid dynamic cardiovascular fluctuations.

The central regulation of cardiovascular activity involves a widespread network of structures ranging from the spinal cord to the cerebral cortex. Previous studies have demonstrated anatomical connections between PSTN neurons and the parasympathetic system.^[^ ^47^ ^]^ However, the exact downstream pathway through which the PSTN exerts parasympathetic output remains unresolved. Early evidence suggested that the PSTN may function as a key relay within a glutamatergic pathway extending from the CeA or insular cortex to the NTS.^[^ ^48^ ^]^ The NTS, located in the medulla oblongata, is a key brainstem region for central cardiovascular regulation. Our anterograde tracing experiments show that PSTN*^Vglut2^* neurons send dense projections throughout the NTS, extending from its rostral to caudal regions. In contrast, projections to the ventrolateral medulla and most subregions of the DMX are sparse, with only modest innervation observed in the rostral DMX. Using whole‐cell patch‐clamp recordings, we identified a monosynaptic projection from PSTN*^Vglut2^* neurons to NTS*^Vglut2^* neurons. These findings suggest that the cardiovascular effects of the PSTN are primarily mediated indirectly via the NTS, rather than through direct modulation of downstream parasympathetic effector brain regions.

Nonspecific presynaptic blockade within the NTS significantly diminishes the heart rate and blood pressure responses elicited by PSTN stimulation.^[^ ^33^ ^]^ Our functional data show that chemogenetic activation of NTS‐projecting PSTN*^Vglut2^* neurons under physiological conditions leads to reductions in heart rate and diastolic blood pressure. Chemogenetic inhibition of these neurons produces cardiovascular effects similar to those observed with global inhibition of PSTN*^Vglut2^* neurons. However, during acute hypertension, the blood pressure‐lowering effect of activating NTS‐projecting PSTN*^Vglut2^* neurons differs from that of activating the broader PSTN*^Vglut2^* population. Specifically, activation of NTS‐projecting neurons does not reduce the peak magnitude of blood pressure elevation but instead accelerates the return to baseline levels. These findings reveal functionally distinct roles of PSTN projections and offer new targets and theoretical insights for the development of non‐pharmacological interventions for hypertension and cardiovascular decompensation.

Although the DMX serves as the primary efferent nucleus for parasympathetic output and the NTS is traditionally recognized as the central hub for visceral afferent processing, substantial evidence indicates that the NTS modulates parasympathetic efferent pathways. For example, photostimulation of NTS neurons projecting to the paraventricular nucleus, lateral parabrachial nucleus, and caudal ventrolateral medulla markedly reduces blood pressure, whereas activation of NTS neurons targeting the rostral ventrolateral medulla evokes a robust hypertensive response accompanied by bradycardia.^[^ ^49^ ^]^ Selective lesioning of NTS neurons disrupts cardiovascular homeostasis by blunting baroreflex sensitivity, increasing blood pressure variability, and inducing arrhythmias.^[^ ^50^ ^]^

Anatomically, parasympathetic preganglionic neurons receive multi‐level projections from several nuclei of the lateral hypothalamic area.^[^ ^51^ ^]^ Parasympathetic preganglionic neurons of the DMV receive neural input from neurons of the NTS*^Vglut2^* neurons.^[^ ^52^, ^53^ ^]^ Through time‐delayed PRV retrograde tracing, Mohanta *et al.* revealed that the NTS likely regulates the artery‐brain circuit via the DMX.^[^ ^41^ ^]^ The NTS receives afferent input from arterial baroreceptors and coordinates autonomic reflexes through projections to both the DMX and Amb, thereby enhancing cardiac parasympathetic output.^[^ ^18^, ^39^ ^]^ These findings collectively confirm the existence of an indirect vagal output pathway mediated by the NTS.

Although our NE‐induced model illuminates acute baroreflex dynamics, these results suggest potential implications for chronic hypertension. It is known that the baroreflex plays a role in long‐term blood pressure control,^[^ ^54^ ^]^ and chronic hypertension may lead to baroreceptor desensitization and baroreflex resetting. In our experiments, PSTN inhibition is associated with sustained hypertension, which may reflect some disturbance in homeostatic regulation. To further explore these acute observations, future research could employ spontaneously hypertensive rats (SHR), a validated model of essential hypertension, to investigate whether sustained PSTN activation mitigates primary hypertension by preserving baroreflex sensitivity. Additionally, chronic angiotensin II infusion models could assess whether PSTN activation prevents target‐organ damage.^[^ ^55^ ^]^ We hypothesize that targeted neuromodulation of the PSTN might help maintain baroreflex sensitivity during prolonged pressure elevation, or could potentially reduce end‐organ damage and inhibit pathological vascular remodeling. This could potentially position PSTN intervention as a novel strategy to disrupt the self‐perpetuating cycle of hypertension.

Beyond its role in cardiovascular homeostasis, our findings uncover a novel facet of PSTN function: the coordinated regulation of locomotor capacity. While prior studies have shown that PSTN influences exploratory behavior through ventral tegmental area and parabrachial nucleus pathways,^[^ ^38^ ^]^ with PSTN*^Crh^* neuron activation enhancing exploration in anxiety‐related paradigms,^[^ ^56^ ^]^ and PSTN*^Vglut2^* neuron manipulation during free‐feeding phases did not significantly affect locomotion.^[^ ^57^ ^]^ However, the specific role of PSTN in regulating voluntary locomotion, particularly movement speed, has remained largely unexplored.

Using voluntary running wheel assays to analyze velocity profiles, our study shows that PSTN activation has little effect on motor performance in mice, whereas inhibition, particularly of PSTN*^Vglut2^* neurons, significantly reduces activity levels and movement speed. We identified a strong negative correlation between PSTN‐modulated MBP fluctuations and key locomotor metrics, including total distance traveled and time spent in fast‐speed locomotion, highlighting the role of PSTN in supporting high‐exertion activities.

We attribute the pronounced locomotor deficits at higher speeds to impaired cardiovascular homeostasis. which significantly hinders high‐intensity locomotion due to diminished hemodynamic responsiveness. This effect was consistently observed across multiple PSTN inhibition methods beyond KA, strongly supporting a cardiovascular basis for the observed deficits. Under physiological conditions, inhibition of either the PSTN or its glutamatergic subgroup increases cardiac output, as measured by the RPP and induces hypertension, imposing sustained myocardial afterload and reducing cardiac efficiency. During high‐intensity exercise, the cardiovascular system, already under stress, struggles to meet elevated hemodynamic demands, resulting in reduced duration of high‐speed locomotion and total distance traveled.

PSTN inhibition also impairs baroreflex function. Normally, the cardiovascular system maintains blood pressure homeostasis through adaptive responses calibrated to exercise intensity. While acute changes in MBP typically do not alter baroreflex sensitivity,^[^ ^39^ ^]^ PSTN inhibition reduces baroreflex sensitivity. This loss of reflex flexibility severely limits the ability of system to adapt to the rapidly increasing circulatory demands of high‐speed movement, further exacerbating motor impairments. Our findings establish the PSTN as a higher‐order center for the baroreflex, likely mediated through the PSTN*^Vglut2^* →NTS circuits. This baroreflex mechanism, is vital for maintaining exercise capacity and may be a therapeutic target for heart failure or autonomic dysfunction.

The contribution of NTS‐projecting PSTN*^Vglut2^* neurons to locomotion is less pronounced than that of direct PSTN manipulation, suggesting the involvement of additional glutamatergic downstream targets in motor control. Additionally, the impact of the PSTN on maximum movement speed in mice appears to be independent of its cardiovascular regulatory functions. Our research indicates that PSTN*^Vglut2^* neurons project downstream to numerous motor‐regulating brain regions, including the basal ganglia, midbrain, pons, and brainstem. These areas are critical for controlling movement speed, direction, and fine motor coordination.^[^ ^24^, ^58^ ^]^ This suggests that the effect of the PSTN on maximum movement speed may be mediated through direct neural pathways rather than solely relying on its regulatory role in the cardiovascular system.

Additionally, PSTN neuronal activation also induces an elevation in pulse pressure. In our study, this increase stemmed primarily from a reduction in diastolic pressure rather than an increase in systolic pressure. Although such pulse pressure elevation has the potential to compromise cardiac efficiency, it did not adversely affect locomotor activity in mice. Current monitoring techniques in our study cannot fully account for this finding. While the tail‐cuff method is a widely accepted and extensively applied approach—demonstrating good correlation with MBP and heart rate measurements obtained via wireless telemetry^[^ ^59^ ^]^—it inevitably induces stress during testing. In contrast, wireless telemetry offers superior advantages for capturing blood pressure and heart rate under more natural physiological conditions, particularly in chronic hypertension models requiring long‐term recordings. Furthermore, telemetry could enable precise synchronization of blood pressure changes with PSTN*^Vglut2^* neuronal calcium signaling dynamics, providing deeper insights into their temporal relationship. To further elucidate how PSTN‐mediated baroreflex influences parasympathetic outputs, intrinsic cardiac function, and broader hemodynamic interactions, future studies should integrate additional methods such as specific ablation of cardiac‐projecting neurons, advanced techniques like wireless telemetry, electrocardiography, echocardiography, and targeted biochemical assays.^[^ ^5^, ^25^, ^41^ ^]^

The PSTN functions as a central hub influencing diverse physiological networks. Although such functional breadth raises concerns about off‐target effects in clinical manipulation, a dialectical perspective can guide targeted interventions: for instance, selective activation of PSTN*^Tac1^* or PSTN*^Vglut2^* subpopulations could suppress eating^[^ ^60^, ^61^ ^]^ and offer therapies for bulimia nervosa or obesity with hypertension, whereas PSTN*^PACAP^* circuits mediating fear‐induced feeding suppression likely serve protective, adaptive roles.^[^ ^57^ ^]^ Crucially, our ongoing work reveals additional PSTN‐mediated effects vital for clinical translation: preliminary data show that PSTN stimulation produces anxiolysis but slows gastrointestinal motility, while PSTN inhibition elevates pain thresholds. These results underscore the profound influence of the PSTN across autonomic, affective, nociceptive, and enteric networks. Therefore, to achieve precise modulation of specific physiological functions and minimize multisystem risks, therapeutic strategies must target functionally specialized PSTN subpopulations and circuits, particularly by leveraging methods such as AAV‐taCasp3‐mediated chemogenetic ablation or other specific vagal‐targeting approaches to refine the PSTN‐vagal efferent pathway.

In summary, our findings establish the PSTN as a critical interoceptive hub that, through its *Vglut2*‐expressing neurons and indirect NTS projections, dynamically regulates baroreflex function to preserve cardiovascular stability and sustain high‐speed locomotion. Chemogenetic activation of these neurons buffers acute hypertensive responses, while their inhibition leads to persistent hypertension, impaired baroreflex sensitivity, and reduced exercise capacity. This work highlights the PSTN as a promising target for neuromodulatory interventions aimed at disrupting the self‐perpetuating cycle of hypertension and supporting physiological resilience.

## 4 Experimental Section

### Ethics Statement

All experimental protocols were approved by the Institutional Animal Care and Use Committee of the Stomatological Hospital of Tongji University (Approval No. 2020‐DW‐13). During all the experiments, we made every effort to minimize the pain and distress experienced by the animals.

### Animals

Adult (8 to 12 weeks old) C57BL/6J male mice (Slac Laboratory Animal, Shanghai), *Vglut2‐Cre* transgenic mice [Slc17a6tm2(Cre)Lowl/J, RRID: 016963, the Jackson Laboratory] and H2B‐GFP mice (Rosa26‐loxp‐STOP‐loxp‐H2B‐GFP, gift of M. He, Fudan University) were used in this study. All of these mice were housed on a 12‐h light/dark cycle under standard conditions in the animal facility with food and water ad libitum unless otherwise noted.

### Stereotaxic Surgery for Drug/Virus Injection and Optic Fiber Implantation

Mice were anesthetized by isoflurane gas anesthesia (induction at 3.5%, and maintenance at 1.5‐2%, AirMac‐DB155), and then placed on a stereotaxic frame (RWD Life Science, Shenzhen, China). After skull exposure, a craniotomy was performed using a micromotor drill. Viruses were delivered via a fine‐glass capillary (Sutter Instrument, 208246) connected to a microliter syringe with Tygon tubing. The syringe was placed on an infusion pump (NANOJET3, Drummond Scientific, USA) for a steady injection rate of 50 nL min^−1^. Target regions included the PSTN (AP: −2.40 mm, ML: ±1.10 mm, DV: ‐5.00 mm from bregma) and NTS (AP: −7.3–7.4 mm, ML: ±0.35 mm, DV: −4.75 mm from bregma). To prevent virus leakage, the injection capillary was left in place for 10 min after the completion of virus injection, and then slowly withdrawn. Finally, the incision was sutured, and the surgical wound was sterilized.

For ablating PSTN neurons, KA (Yuanye Bio‐Technology, Cat#S30773) was dissolved in saline to a concentration of 2 µg/µL and bilaterally injected into the PSTN (40 nL per side). Behavioral assays were conducted three days post‐injection.

For chemogenetic modulation of PSTN neurons, rAAV‐hSyn‐hM3D(Gq)‐EGFP‐WPRE‐hGH PolyA, AAV2/9 (>5.00E+12 GC mL^−1^) or PAAV‐hSyn‐HA‐hM4D(Gi)‐mCherry‐WPRE, AAV2/9 (1.38E+13 GC mL^−1^) or PAAV‐hSyn‐mCherry‐3 × FLAG‐WPRE, AAV2/9 (1.28E+13 GC mL^−1^) was injected bilaterally into the PSTN 50 nL for each side of wild type mice. Experiments were performed three weeks post‐injection.

For monitoring PSTN*^Vglut2^* neurons via fiber photometry, 50 nL of rAAV‐hSyn‐DIO‐GCaMP6s, AAV2/9 (3.02E+12 GC mL^−1^) was injected unilaterally into the PSTN of *Vglut2‐cre* mice. Optical fiber implants [200 µm in diameter, numerical aperture (NA) = 0.37] was implanted above the PSTN (0.1 mm above virus injection coordinate). Two screws were implanted away from the injection site as anchors. Superglue and dental cement were used to fix the implantation. Experiments were conducted three weeks post‐injection.

For chemogenetic modulation of PSTN*^Vglut2^* neurons, rAAV‐hSyn‐DIO‐hM3D(Gq)‐EGFP, AAV2/9 (5.39E+12 GC mL^−1^) or rAAV‐hSyn‐DIO‐hM4D(Gi)‐P2A‐EGFP, AAV2/9 (5.01E+12 GC mL^−1^) or rAAV‐hSyn‐DIO‐EGFP, AAV2/9 (5.05E+12 GC mL^−1^) was injected bilaterally into the PSTN 50 nL for each side of *Vglut2‐cre* mice. Experiments took place three weeks after injection.

For determining the outputs of PSTN*^Vglut2^* neurons, 50 nL of rAAV‐hSyn‐DIO‐EGFP, AAV2/9 (5.05E+12 GC mL^−1^) was unilateralinjected into the PSTN of *Vglut2‐Cre* transgenic mice. Mice were sacrificed four weeks after the injection.

To label NTS‐projecting PSTN*^Vglut2^* neurons, AAV2/2Retro‐CAG‐FLEX‐FlpO‐WPRE‐PA (1.89E+13 GC mL^−1^) was injected bilaterally into the NTS 100 nL for each side, and rAAV‐nEF1a‐fDIO‐EGFP‐WPRE‐hGH PolyA was injected bilaterally into the PSTN 50 nL for each side of *Vglut2‐Cre* mice.

For chemogenetic modulation of NTS ‐projecting PSTN*^Vglut2^* neurons, AAV2/2Retro‐CAG‐FLEX‐FlpO‐WPRE‐PA (1.89E+13 GC mL^−1^) was injected bilaterally into the NTS 100 nL for each side of *Vglut2‐Cre* mice, and rAAVnEF1a‐fDIO‐hM3D(Gq)‐EGFP‐WPRE‐hGH polyA, AAV2/9 (5.57E+12 GC mL^−1^) or rAAVnEF1a‐fDIO‐hM4D(Gi)‐EGFP‐WPRE‐hGH polyA, AAV2/9 (>5.00E+12 GC mL^−1^) or rAAVnEF1a‐fDIO‐EGFP‐WPRE‐hGH polyA, AAV2/9 (>5.00E+12 GC mL^−1^) into the PSTN 50 nL for each side of *Vglut2‐Cre* mice. Experiments were conducted four weeks post‐injection.

### Histology

On completion of the calcium recording, chemogenetic experiments, and after CNO administration (chemogenetic), animals were deeply anesthetized by sodium pentobarbital and transcardially perfused with 30 mL 0.1 m phosphate‐buffered saline (PBS) followed by 50 mL 4% paraformaldehyde (PFA) in PBS. After removal, brains were fixed for 24 h in 4% PFA, and then incubated in 30% sucrose in PBS solution at 4 °C until they sank. Brains were embedded in OCT compound and then sliced into three series of 30‐µm sections on a freezing microtome (CM1850, Leica, Germany).

For fluorescent staining of c‐Fos, free‐floating sections were first blocked in 0.01 m PBS solution containing 10% NGS, 1% BAS and 0.3% Triton‐X, and then incubated with primary antibody (rabbit anti‐Fos, SYSY system, 226008, 1:5000, RRID:AB_2891278; guinea pig anti‐Fos, SYSY system, 226004, 1:5000, RRID:AB_2619946) at 4 °C for 12 h. Sections were then washed with PBS for three times and incubated with secondary antibody (Cy3‐conjugated affiniPure donkey anti‐guinea pig, Jackson Immuno Research, 1:800, RRID:AB_2340460; Alexa Fluor 647 goat anti‐rabbit, Jackson Immuno Research, 1:800, RRID:AB_2492288) for 2 h at room temperature.

For fluorescent detection of NeuN, sections were washed in blocking buffer, and incubated in primary antibody (NeuN, 1:1000, Millipore, MAB377, RRID: AB_2298772) for 1 h at room temperature and and then incubated at 4°C for 12‐ h. Sections were then washed with PBS for three times and incubated in secondary antibody (Cy^TM^3‐conjugated affiniPure donkey anti‐guinea pig, Jackson Immuno Research, 1:800, RRID: AB_2340460) at room temperature for 2‐h. Then, sections were washed three times in PBS and mounted on slides with Fluoromout‐GTM (CITOTEST).

For hoechst staining, prepare the Hoechst 33342 (Cell Signaling Technology, 4082, RRID: AB_10626776) dye stock solution by dissolving the contents of one vial (100 mg) in 10 mL of deionized water (ddH_2_O) to create a 10 mg mL^−1^ (16.23 mm) solution. Incubate the cells with Hoechst (100 ng mL^−1^ in PBS) in the dark for 5 min at room temperature.

### Imaging

Fluorescence images, including the verification of virus expression and fiber implantation, were acquired on a Leica SP8 confocal microscope or a Leica DM6B microscope using 10 × and 20 × objectives. Imaging settings were kept consistent for all groups. And all cell counting was carried out manually offline in a blind manner with LAS X 1.4.7 (Leica microsystems, Germany).

### Electrophysiological Recordings—Brain Slice Preparation

Mice were deeply anesthetized with pentobarbital sodium and transcardially perfused with ice‐cold oxygenated cutting solution (pH 7.3–7.4; 300–305 mOsm L^−1^) containing the following components (in mM): 93 N‐methyl‐d‐glucamine, 2.5 KCl, 1.2 NaH_2_PO_4_, 30 NaHCO_3_, 10 MgSO_4_, 0.5 CaCl_2_·H_2_O, 20 HEPES, 25 D‐glucose, 5 Na‐ascorbate, 2 thiourea, and 3 Na‐pyruvate (ph 7.3–7.4;300–310 mOsm L^−1^). Coronal slices (300 µm) encompassing the PSTN or NTS regions were prepared using a vibrating microtome (VT1200s, Leica), followed by a sequential incubation protocol: initial stabilization in carbogen‐saturated cutting solution at 32 ± 0.5 °C for 12 min, then incubation for at least 1 h in oxygenated ACSF solution maintained at the same temperature, with the ACSF composition consisting of (in mM): 126 NaCl, 3 KCl, 1.2 NaH_2_PO_4_, 1.3 MgCl_2_·6H_2_O, 2.4 CaCl_2_·2H_2_O, 26 NaHCO_3_, and 10 glucose (PH 7.3 – 7.4; 300–310 mOsm L). After incubation, slices were held under continuous carbogenation at room temperature for 1 h prior to being transferred to a slice chamber, where they were continuously perfused with oxygenated ACSF (2 ml/min) at 32 °C during electrophysiological recordings.

### Whole‐Cell Patch‐Clamp Recordings

Target region neurons were visualized using an upright microscope (BX51WI, Olympus) with 40 × water‐immersion objective, equipped with infrared differential interference contrast (IR/DIC) optics and an infrared camera (DAGE‐MTI) connected to a video monitor. Whole‐cell patch‐clamp recordings were performed on visually identified neurons in the PSTN and NTS using borosilicate glass pipettes (1.5 mm outer diameter; VitalSense Scientific Instruments) pulled with a five‐stage horizontal puller (P2000, Sutter Instruments) to 4–8 MΩ resistance. For CNO testing, the potassium gluconate‐based internal solution contained (in mM): 126 potassium gluconate, 2 KCl, 10 HEPES, 2 MgCl_2_, 0.2 EGTA, 2.5 Mg‐ATP, and 0.25 Na_3_GTP (pH 7.3; 280–290 mOsm L^−1^). Cs intersolution used for light contained (in mM): 120 CsMeSO_3,_ 15 CsCl, 8 NaCl, 10TEA‐Cl, 10 HEPES, 0.2 EGTA, 4 Mg‐ATP, 0.3 Na_3_GTP, 5 QX314‐Cl. Current‐evoked firing patterns were recorded in current‐clamp mode following at least 5 min for neuronal stabilization, with all experiments conducted at 28–30 °C under continuous perfusion.

### CNO‐Evoked Response

To validate the DREADDs function, current‐clamp recordings were conducted at a resting membrane potential from Gq or Gi expressing PSTN neurons using 4–8 MΩ glass pipettes filled with standard K^+^ intersolution. Electrophysiological signals were acquired through a MultiClamp 700B amplifier (Axon Instruments, USA) operating in whole‐cell configurations, with current‐clamp experiments. All recordings were conducted at 28–30 °C with signal conditioning comprising 10 kHz low‐pass filtering and 10 kHz digitization using a DigiData 1440A system (Axon Instruments, USA). Data acquisition and analysis were executed through pCLAMP 10.6 software suite (Axon Instruments, USA), with neuronal responses quantified after establishing stable baseline parameters.

### Light‐Evoked Response

To verify the functional properties of the AAV‐DIO‐ChR2‐mCherry virus, 50 nL of the virus was stereotaxically injected into the PSTN of Vglut2::H2B mice. ChR2‐expressing Vglut2 neurons in the PSTN, tagged with mCherry, were visualized using fluorescence microscopy and stimulated with blue laser light (473 nm) at 5 Hz and 10 Hz, with a 5 ms pulse width and 5–10 mW power. In NTS brain slices, blue light pulses (473 nm, 5 ms duration) were applied to ChR2‐expressing fibers from PSTN*^Vglut2^* neurons. Light‐evoked excitatory postsynaptic currents (EPSCs) were recorded from EGFP‐labeled NTS^Vglut2^ neurons under a holding potential of −70 mV. These EPSCs were blocked by 10 µM NBQX (Tocris, USA) in the ACSF. To confirm that the recorded currents in NTS*^Vglut2^* neurons originated from direct monosynaptic connections, 1 µM TTX (TaiZhou KangTe) and 1 µM 4‐AP (Tocris, USA) were added to the ACSF to block action potential‐dependent synaptic transmission, followed by repeated optical stimulation and current recording. Data were acquired using Clampex10.6 (Axon Instruments, USA), with signals amplified, low‐pass‐filtered at 2 kHz, and digitized at a sampling rate of 10 kHz.

### Tail‐Cuff Plethysmography and Experimental Design

All tests were conducted in a dimly lit and quiet room. Animals were acclimated to the testing environment for at least 2 h before the start of formal testing. Heart rate, SBP, DBP and MBP. were measured in awake mice using a non‐invasive computerized tail‐cuff system (BP‐2010A, Softron, Japan) based on photoplethysmographic infrared sensing technology. Measurements were obtained before and after administration of clozapine‐N‐oxide (CNO; Cat# A3317, APExBIO, USA) or norepinephrine (NE; Cat# B25875, Yuanye Bio‐Technology, China). Prior to formal testing, mice underwent a two‐day habituation period to the testing bag, during which they were trained to voluntarily enter the bag within 2–3 min. This procedure ensured that all mice had a consistent resting period inside the testing bag from the time of entry until the first measurement, which was taken 10 min after CNO injection.^[^ ^62^, ^63^, ^64^, ^65^ ^]^ After habituation to the testing bag, mice were subjected to three days of adaptive tail‐cuff training.^[^ ^59^ ^]^ A baseline measurement was recorded one day after the last habituation. MBP was calculated as one‐third of SBP plus two‐thirds of DBP. For each mouse, heart rate and blood pressure at each time point were recorded as the average of three consecutive measurements.

### Wheeling Running Test

Voluntary running wheel activity was assessed using polycarbonate cages (20 cm × 32 cm × 14 cm) equipped with stainless steel running wheels (28 cm diameter, 6.7 cm width), designed for bidirectional rotation. Multiple cages were housed in a controlled testing environment, with each wheel connected to a computer system that recorded distance and speed automatically using the Trigger Master System (Fanbi Intelligent Technology, Shanghai). To minimize external influences, assessments were conducted without experimenters present.

Mice were acclimated to the setup through daily 1‐h sessions in the running wheel cage for at least three consecutive days. Baseline running measurements were recorded the day after the final habituation session. Movement speeds were categorized as follows: high‐speed locomotion (speed > 15 cm s^−1^), medium‐speed locomotion (speed > 10 cm s^−1^), slow‐speed locomotion (speed > 5 cm s^−1^), and immobility (speed < 1 cm s^−1^). During the 12‐h running wheel test, mice had unrestricted access to the wheel. In contrast, the 1‐h running wheel test, defined as semi‐voluntary, included a barrier at the exit of the wheel to prevent mice from leaving. All running wheel tests commenced 20 min after CNO injection.

### Data Analysis for PSTN*^Vglut2^* Neuron Output

After sectioning and Hoechst staining, one section was selected from every five 40‐µm‐thick slices for analysis. The mean intensity values of PSTN^Vglut2^ neuron projection axons were measured using the LAS X intensity stack profile tool. Background fluorescence was subtracted, and the projection intensity ratio was determined by dividing the mean intensity of the projection by the mean intensity of the PSTN. All ratios were normalized for consistent comparison across samples. Brain regions were identified using the Allen Brain Reference Atlas (http://atlas.brain‐map.org/) and the Paxinos and Franklin Mouse Brain Atlas.^[^ ^66^ ^]^

### Statistics

Data are presented as the mean ± standard error of the mean (SEM). Statistical analyses were performed using Student's t‐test for comparisons between two groups, with significance levels denoted as *p < 0.05, **p < 0.01, ***p < 0.001, and ****p ＜ 0.0001. For experiments involving multiple groups, one‐way was employed and two‐way ANOVA followed by Bonferroni post hoc analyses. The n used for these analyses represents the number of mice. Graph generation and statistical analyses were conducted using GraphPad Prism 10 (GraphPad Software, USA). Electrophysiological data were analyzed offline using Clampfit software 10.7 (Axon Instruments, USA). Adobe Illustrator 2025 (Adobe, USA) was used for final figure assembly and statistical annotation. All statistical details, including significance results, the number of individual experiment n, and other relevant information for data comparison, are provided in Table S1 (Supporting Information).

## Conflict of Interest

The authors declare no conflict of interest to report.

## Supporting information



Supporting Information

## Data Availability

Research data are not shared.
