# Supplementary material for: Parasubthalamic Glutamatergic Neurons Coordinate Cardiovascular Homeostasis and Locomotion in Mice
Source: Adv Sci (Weinh). 2025 Jul 28;12(40):e17353. doi: 10.1002/advs.202417353 (PMC12561448; doi:10.1002/advs.202417353)
Supplement: Supplementary file 1 — Supporting Information [file ADVS-12-e17353-s001.docx]

Supporting Information

**Parasubthalamic Glutamatergic Neurons Coordinate Cardiovascular Homeostasis and Locomotion in Mice**

*Ming-Xuan Lu, Jin-Yin Huang, Xin-Zhe Xu, Ji-Yu Sun, Dan-Ni Zou, Jia-Yao Zhang, Chen Chi, Qi Zhang*, Wei-Cai Liu**


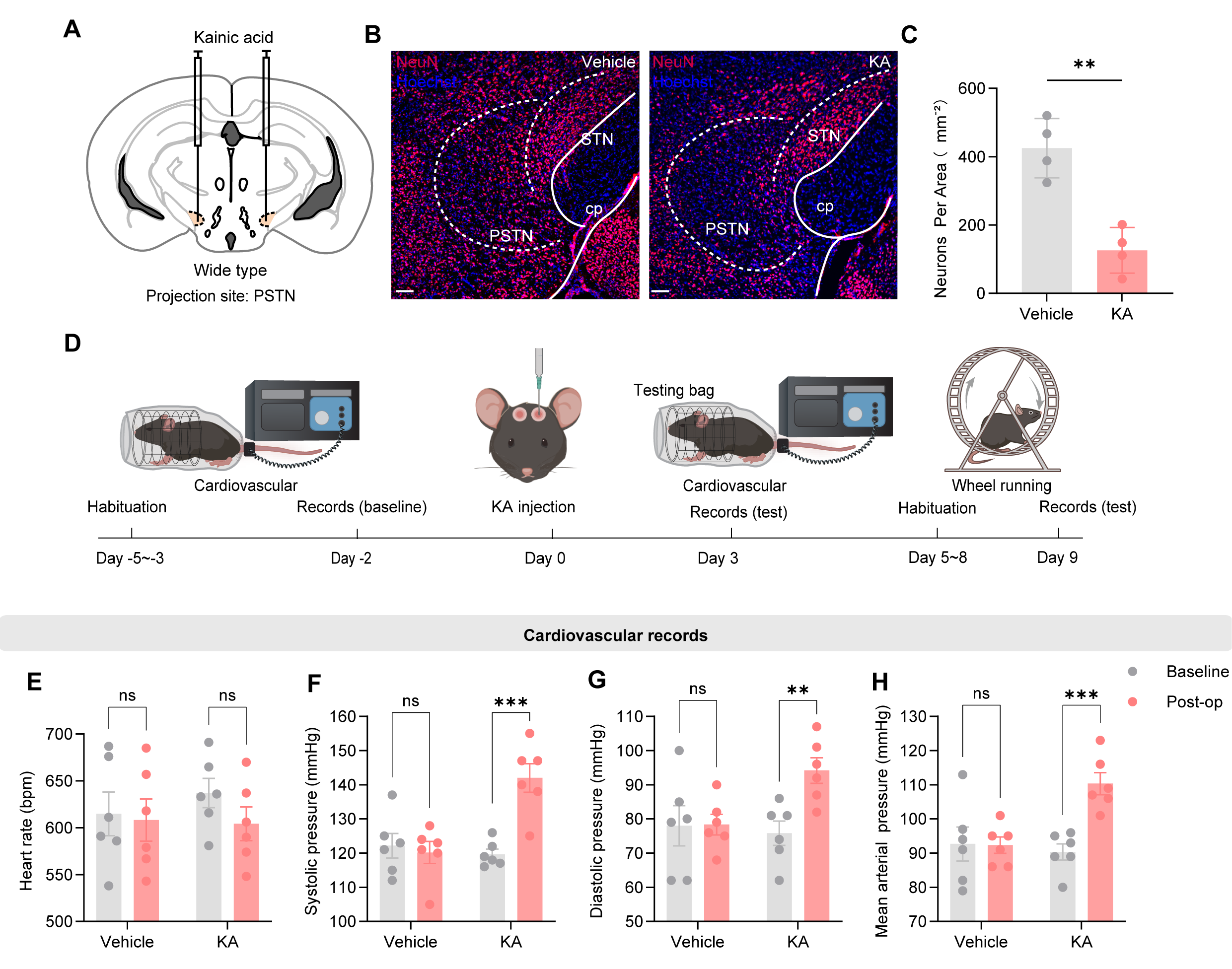
Figure S1. PSTN Neurons Ablation Increases Blood Pressure and Impairs Locomotion.

A) Schematic of microinjection into the PSTN of the C57BL/6J mice. B) Representative images showing KA induced PSTN neurons ablation at the injection site, NeuN (red), Hoechst (blue). PSTN: parasubthalamic nucleus. STN: subthalamic nucleus. cp: cerebral peduncle. Scale bar: 100 µm. C) Quantification of NeuN in the PSTN (Vehicle n =4, KA n =4, unpaired two-sided t-test; t_6_ = 5.458, *p* = 0.0016) D) Schematic of the protocol in experiments. E) Summary of heart rate (Two-way ANOVA Interaction: not statistically significant). F) Summary of systolic pressure (Two-way ANOVA Interaction: F_1,20_ = 13.70, *p* =0.0014; KA baseline vs KA Post-po, *p* = 0.0001,). G) Diastolic pressure, Two-way ANOVA Interaction: F_1,20_ = 4.591, *p* = 0.046; KA baseline vs KA Post-po, *p* = 0.0058. H) Mean pressure, Two-way ANOVA Interaction: F_1,20_ = 8.915, *p* =0.0073; KA baseline vs KA Post-po, *p* = 0.0011) . Post-po: post KA operation. Each circle represents results from one mouse, n = 6. Two-way ANOVA with fisher LSD analysis. All data are presented as means ± SEM. ns, *p*> 0.05; **p*< 0.05; ***p*< 0.01 and ****p*< 0.001. See also Table S1.

**
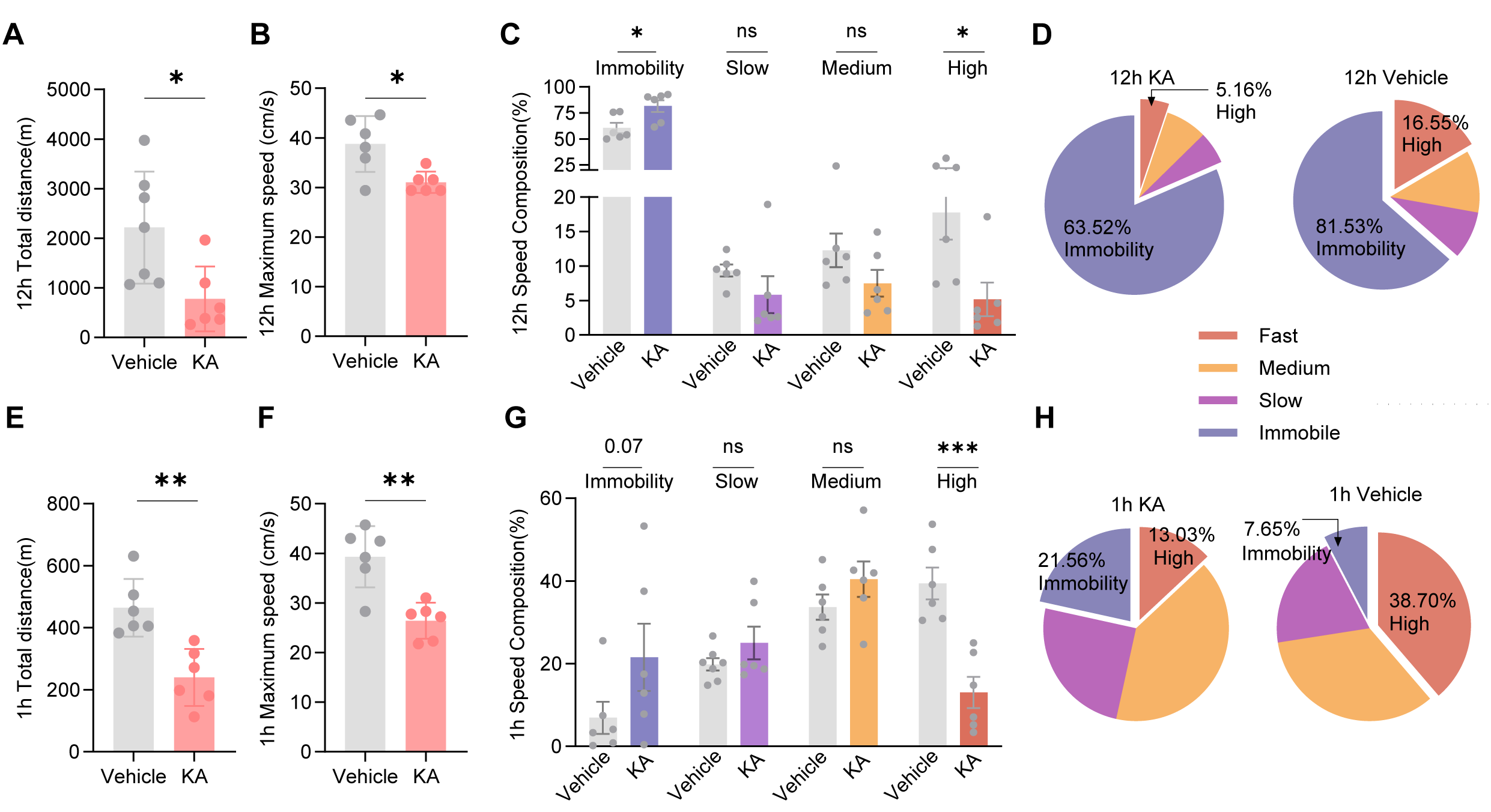
**Figure S2. PSTN Neurons Ablation Impairs Locomotion.

A,B) Summary of locomotion in 12-hour free wheel running in mice (n = 6; unpaired two-sided t-test; Total distance: t_10_= 2.958, P = 0.0143; Maximum speed: t_10_ = 3.141, P = 0.0105).C) Summary of 12-hour speed composition in mice (n = 6; unpaired two-sided t-test; Immobility: t_10_= 2.848, P = 0.0173; High-speed locomotion time: t_10_ = 2.711, P = 0.0219). D) Pie charts showing 12-hour speed composition in the KA and vehicle groups. E, F) Summary of locomotion in 1-hour semi-free wheel running in mice (n = 6; unpaired two-sided t-test; Total distance: t_10_= 4.263, P = 0.0017; Maximum speed: t_10_ =4.371, P = 0.0014). G) Summary of 1-hour speed composition in mice (n = 6; unpaired two-sided t-test; Immobility: t_10_= 2.025, P = 0.07; High-speed locomotion time: t_10_ = 4.867, P = 0.0007). H) Pie charts showing 1-hour speed composition in the KA and vehicle groups. Each circle represents results from one mouse. All data are presented as means ± SEM. ns, *p*> 0.05; **p*< 0.05; ***p*< 0.01 and ****p*< 0.001. See also Table S1.


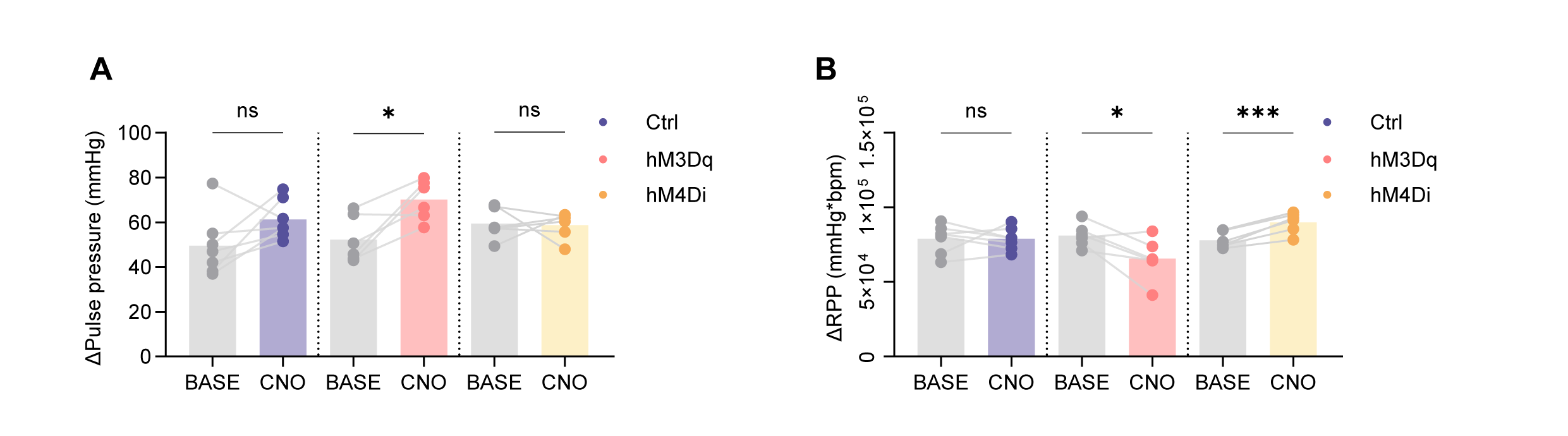
Figure S3. Effects of Manipulating PSTN on Pulse Pressure and RPP.

A) Summary of mean ∆pulse pressure in C57BL/6J mice within 1-hour after CNO administration, paired two-sided t-test; mCherry mice: n = 7, not statistically significant; hM3Dq mice: n = 6, t_5_ = 3.486, *p* = 0.0175; hM4Di mice: n = 6; not statistically significant. B) Summary of mean ∆RPP in C57BL/6J mice within 1-hour after CNO administration, ∆RPP, mCherry mice: n = 7, not statistically significant; hM3Dq mice: n = 6, t_5_ = 2.842, *p* = 0.0362; hM4Di mice: n = 6; t_5_ = 7.003, *p* = 0.0009). Δ represents the amount of change in cardiovascular parameters relative to baseline. Each circle represents results from one mouse. ns, *p*> 0.05; **p*< 0.05; ***p*< 0.01 and ****p*< 0.001. See also Table S1.


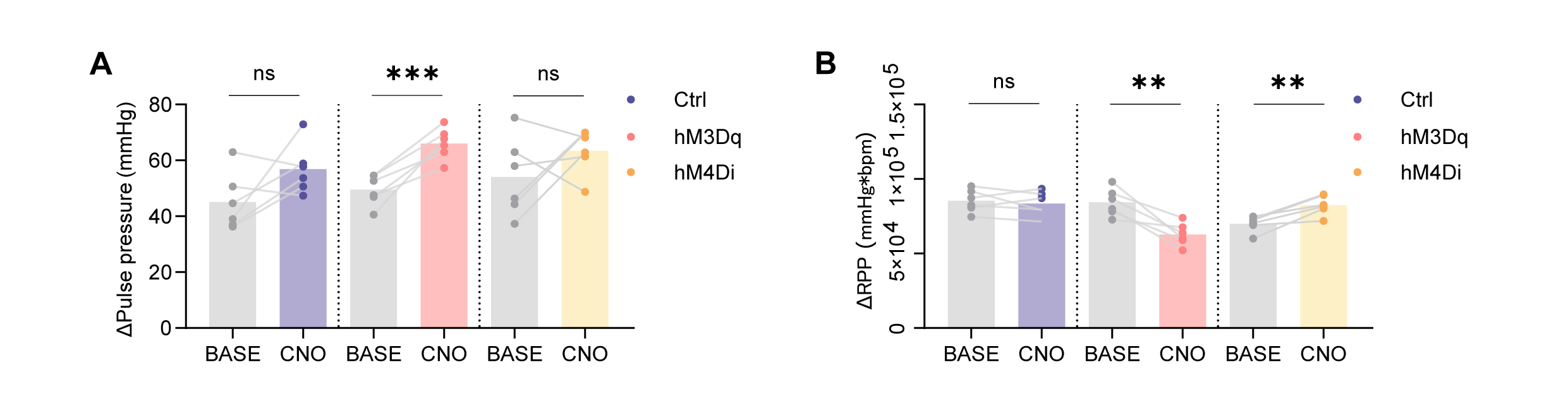
Figure S4. Effects of Manipulating PSTN*^Vglut2^* Neurons on Pulse Pressure and RPP.

A) Summary of mean ∆pulse pressure in *Vglut2-Cre* mice within 1-hour after CNO administration, (paired two-sided t-test; EGFP mice: n = 6, not statistically significant; hM3Dq mice: n = 6, t_5_ = 6.256, *p* = 0.0015; hM4Di mice: n = 6; not statistically significant). B) Summary of mean ∆RPP in *Vglut2-Cre* mice within 1-hour after CNO administration, (paired two-sided t-test; EGFP mice: n = 6, not statistically significant; hM3Dq mice: n = 6, t_5_ = 4.793, *p* = 0.0049; hM4Di mice: n = 6; t_5_ = 4.758, *p* = 0.0051). Δ represents the amount of change in cardiovascular parameters relative to baseline. Each circle represents results from one mouse. ns, *p*> 0.05; **p*< 0.05; ***p*< 0.01. See also Table S1.


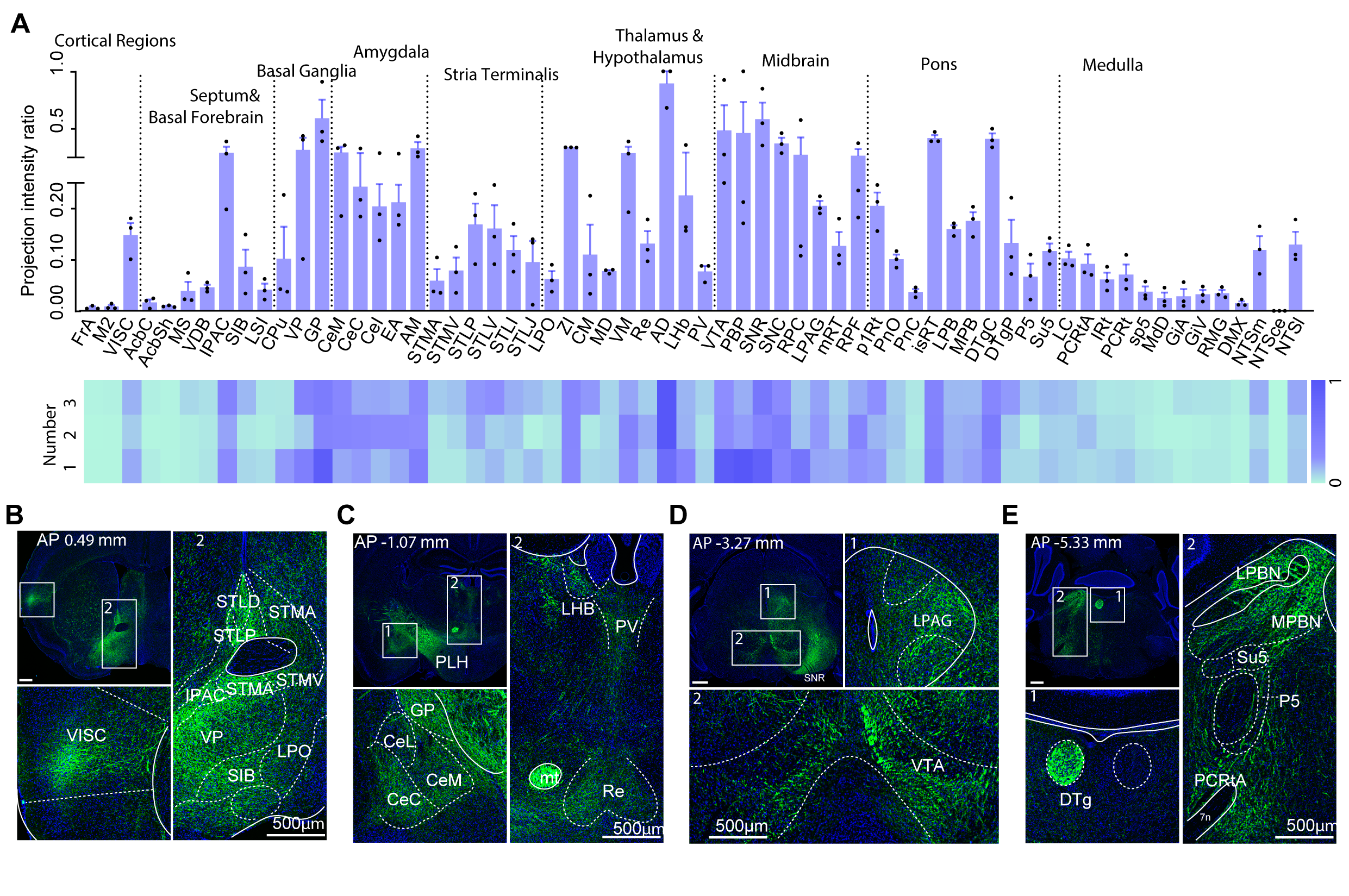
Figure S5. Downstream Targets of PSTN*^Vglut2^* Neurons.

A) Summary of whole-brain outputs from PSTN*^Vglut2^* neurons. Quantitative analysis of EGFP-labeled axon terminal intensity across 64 brain regions in individual PSTN-injected cases (n=3). B-E) Representative EGFP-labeled axon terminal structures of PSTN*^Vglut2^* neurons. Scale bar: 500 µm. Each circle represents results from one mouse. Data are represented as mean ± SEM. Projection intensity ratio calculations are detailed in Method 4.12.


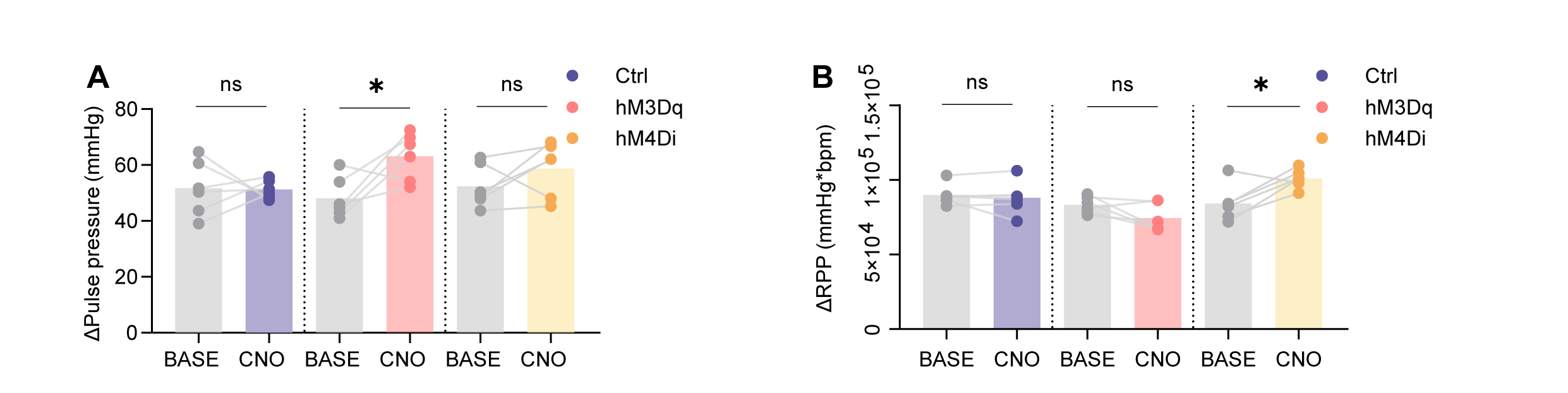
Figure S6. Effects of Manipulating NTS-projecting PSTN*^Vglut2^* Neurons on Pulse Pressure and RPP.

A,B) Summary of mean pulse pressure changes in *Vglut2-Cre* mice within 1-hour after CNO administration (∆pulse pressure, paired two-sided t-test; EGFP mice: n=6, not statistically significant; hM3Dq mice: n=6, t_5_=2.876, *p =* 0.0348; hM4Di mice: n=6; not statistically significant. ∆RPP, EGFP mice: n=6, not statistically significant; hM3Dq mice: n=6, t_5_=2.113, *p =* 0.0883; hM4Di mice: n=6; t_5_=3.092, *p =* 0.0271). Δ represents the amount of change in cardiovascular parameters relative to baseline. Each circle represents results from one mouse. ns, *p*> 0.05; **p*< 0.05. See also Table S1.

Table S1. Extended statistical information for Figures 1-8, and Figures S1-S6.

| Supplementary Table S1 | | | | | | | | | | | |
| --- | --- | --- | --- | --- | --- | --- | --- | --- | --- | --- | --- |
| Extended statistical information for Figures 1-8, and Figures S1-S6. | | | | | | | | | | | |
| Figure | Experiment | Sample size | | | | | | | Analysis | P value | t/F/r  value |
| Fig.1 | | | | | | | | | | | |
|  | CNO |  | | | |  | | | Two-way RM ANOVA, Bonferroni MC |  |  |
| Fig.1E | △HR | mCherry (7) | | | | hM3Dq (6) | | | 10-65min | 0.0140 | F (1, 11) = 8.504 |
| Fig.1F | △SBP | mCherry (7) | | | | hM3Dq (6) | | | 10-65min | 0.2098 | F (1, 11) = 1.774 |
| Fig.1G | △HR | mCherry (7) | | | | hM4Di (6) | | | 10-65min | 0.0637 | F (1, 11) = 4.249 |
| Fig.1H | △SBP | mCherry (7) | | | | hM4Di (6) | | | 10-65min | 0.0153 | F (1, 11) = 8.224 |
| Fig.1I | △DBP | mCherry (7) | | | | hM3Dq (6) | | | 10-65min | 0.9520 | F (1, 11) = 0.003785 |
| Fig.1J | △MBP | mCherry (7) | | | | hM3Dq (6) | | | 10-65min | 0.7219 | F (1, 11) = 0.1334 |
| Fig.1K | △DBP | mCherry (7) | | | | hM4Di (6) | | | 10-65min | 0.0011 | F (1, 11) = 19.36 |
| Fig.1L | △MBP | mCherry (7) | | | | hM4Di (6) | | | 10-65min | 0.0007 | F (1, 11) = 21.39 |
|  | CNO+NE |  | | | |  | | | Two-way RM ANOVA, Bonferroni MC |  |  |
| Fig.1M | △HR | mCherry (7) | | | | hM3Dq (7) | | | 10-65min | 0.0042 | F (1, 12) = 12.42 |
| Fig.1N | △SBP | mCherry (7) | | | | hM3Dq (7) | | | 10-65min | 0.0026 | F (1, 12) = 14.30 |
| Fig.1O | △HR | mCherry (7) | | | | hM4Di (7) | | | 10-65min | 0.9389 | F (1, 12) = 0.006128 |
| Fig.1P | △SBP | mCherry (7) | | | | hM4Di (7) | | | 10-65min | 0.0278 | F (1, 12) = 6.266 |
| Fig.1Q | △DBP | mCherry (7) | | | | hM3Dq (7) | | | 10-65min | 0.5888 | F (1, 12) = 0.3086 |
| Fig.1R | △MBP | mCherry (7) | | | | hM3Dq (7) | | | 10-65min | 0.1893 | F (1, 12) = 1.936 |
| Fig.1S | △DBP | mCherry (7) | | | | hM4Di (7) | | | 10-65min | 0.0300 | F (1, 12) = 6.055 |
| Fig.1T | △MBP | mCherry (7) | | | | hM4Di (7) | | | 10-65min | 0.0102 | F (1, 12) = 9.255 |
| Fig.2 | | | | | | | | | | | |
| Fig.2A | Total distance | mCherry BASE (7) | | | | mCherry CNO (7) | | | Paired two-sided t-test | 0.3539 | t=1.005, df=6 |
|  |  | hM3Dq BASE (7) | | | | hM3Dq CNO (7) | | |  | 0.3195 | t=1.085, df=6 |
|  |  | hM4Di BASE (6) | | | | hM4Di CNO (6) | | |  | 0.0039 | t=5.065, df=5 |
| Fig.2B | Maximum  speed | mCherry BASE (7) | | | | mCherry CNO (7) | | | Paired two-sided t-test | 0.6023 | t=0.5498, df=6 |
|  |  | hM3Dq BASE (7) | | | | hM3Dq CNO (7) | | |  | 0.4778 | t=0.7568, df=6 |
|  |  | hM4Di BASE (6) | | | | hM4Di CNO (6) | | |  | 0.0043 | t=4.944, df=5 |
| Fig.2C | Speed composition |  | | | | | | |  |  |  |
|  | High-speed | mCherry (7) | | | | | hM3Dq (7) | | One-way ANOVA | 0.4213 | F (2, 17) = 13.49 |
|  |  | mCherry (7) | | | | | hM4Di (6) | |  | 0.0030 |  |
|  | Medium-speed | mCherry (7) | | | | | hM3Dq (7) | |  | 0.9068 | F (2, 17) = 3.014 |
|  |  | mCherry (7) | | | | hM4Di (6) | | |  | 0.0616 |  |
|  | Slow-speed | mCherry (7) | | | | hM3Dq (7) | | |  | 0.0489 | F (2, 17) = 21.31 |
|  |  | mCherry (7) | | | | hM4Di (6) | | |  | 0.0013 |  |
| Fig.2 | Correlation |  |  | | | | |  |  |  |  |
| Fig.2E | Total distance vs \|ΔMBP\| | mCherry (7) | hM3Dq (6) | | | | | hM4Di (6) | Linear regression (Pearson) | 0.0348 | r= -0.4861 |
| Fig.2F | High-speed locomotion vs \|ΔMBP\| | mCherry (7) | hM3Dq (6) | | | | | hM4Di (6) |  | 0.0387 | r= -0.4775 |
| Fig.2G | Maximum speed vs \|ΔMBP\| | mCherry (7) | hM3Dq (6) | | | | | hM4Di (6) |  | 0.1018 | r= -0.3869 |
| Fig.3 | | | | | | | | | | | |
| Fig.3B | c-Fos+ cells | Saline (4) | | | | NE (4) | | | Unpaired two-sided t-test | <0.0001 | t=9.935, df=6 |
| Fig.3H | AUC | Saline (6) | | | | NE (6) | | | Paired two-sided t-test | 0.0114 | t=3.094, df=10 |
| Fig.4 | | | | | | | | | | | |
|  | CNO |  | | | |  | | | Two-way RM ANOVA, Bonferroni MC |  |  |
| Fig.4E | △HR | EGFP (7) | | | | hM3Dq (6) | | | 10-65min | <0.0001 | F (1, 11) = 45.69 |
| Fig.4F | △SBP | EGFP (7) | | | | hM3Dq (6) | | | 10-65min | 0.8550 | F (1, 11) = 0.03498 |
| Fig.4G | △HR | EGFP (7) | | | | hM4Di (6) | | | 10-65min | 0.6220 | F (1, 11) = 0.2573 |
| Fig.4H | △SBP | EGFP (7) | | | | hM4Di (6) | | | 10-65min | 0.0065 | F (1, 11) = 11.24 |
| Fig.4I | △DBP | EGFP (7) | | | | hM3Dq (6) | | | 10-65min | 0.2947 | F (1, 11) = 1.211 |
| Fig.4J | △MBP | EGFP (7) | | | | hM3Dq (6) | | | 10-65min | 0.4234 | F (1, 11) = 0.6913 |
| Fig.4K | △DBP | EGFP (7) | | | | hM4Di (6) | | | 10-65min | 0.0013 | F (1, 11) = 18.42 |
| Fig.4L | △MBP | EGFP (7) | | | | hM4Di (6) | | | 10-65min | <0.0001 | F (1, 11) = 35.06 |
|  | CNO+NE |  | | | |  | | | Two-way RM ANOVA, Bonferroni MC |  |  |
| Fig.4M | △HR | EGFP (6) | | | | hM3Dq (6) | | | 10-65min | 0.2000 | F (1, 10) = 1.883 |
| Fig.4N | △SBP | EGFP (6)  hM3Dq (6) | | | | | | | 10-65min | 0.7560 | F (1, 10) = 0.1020 |
|  |  |  |  |  |  |  |  |  | 10min | 0.0001 |  |
|  |  |  |  |  |  |  |  |  | 15min | 0.0193 |  |
|  |  |  |  |  |  |  |  |  | 20min | 0.0047 |  |
| Fig.4O | △HR | EGFP (6) | | | | hM4Di (6) | | | 10-65min | 0.1076 | F (1, 10) = 3.125 |
| Fig.4P | △SBP | EGFP (6) | | | | hM4Di (6) | | | 10-65min | 0.0029 | F (1, 10) = 15.33 |
| Fig.4Q | △DBP | EGFP (6)  hM3Dq (6) | | | | | | | 10-65min | 0.1621 | F (1, 10) = 2.278 |
|  |  |  |  |  |  |  |  |  | 10min | 0.0123 |  |
|  |  |  |  |  |  |  |  |  | 15min | 0.0093 |  |
|  |  |  |  |  |  |  |  |  | 20min | 0.0009 |  |
| Fig.4R | △MBP | EGFP (6)  hM3Dq (6) | | | | | | | 10-65min | 0.2524 | F (1, 10) = 1.476 |
|  |  |  |  |  |  |  |  |  | 10min | 0.0010 |  |
|  |  |  |  |  |  |  |  |  | 15min | 0.0057 |  |
|  |  |  |  |  |  |  |  |  | 20min | 0.0004 |  |
| Fig.4S | △DBP | EGFP (6) | | | | hM4Di (6) | | | 10-65min | 0.0169 | F (1, 10) = 8.193 |
| Fig.4T | △MBP | EGFP (6) | | | | hM4Di (6) | | | 10-65min | 0.0041 | F (1, 10) = 13.66 |
| Fig.5 | | | | | | | | | | | |
| Fig.5A | Total distance | EGFP BASE (6) | | | | EGFP CNO (6) | | | Paired two-sided t-test | 0.3166 | t=1.112, df=5 |
|  |  | hM3Dq BASE (6) | | | | hM3Dq CNO (6) | | |  | 0.0461 | t=2.637, df=5 |
|  |  | hM4Di BASE (6) | | | | hM4Di CNO (6) | | |  | 0.0002 | t=10.13, df=5 |
| Fig.5B | Maximum  speed | EGFP BASE (6) | | | | EGFP CNO (6) | | | Paired two-sided t-test | 0.2322 | t=1.359, df=5 |
|  |  | hM3Dq BASE (6) | | | | hM3Dq CNO (6) | | |  | 0.0645 | t=2.363, df=5 |
|  |  | hM4Di  BASE (6) | | | | hM4Di CNO (6) | | |  | 0.0004 | t=8.298, df=5 |
| Fig. 5C | Speed composition |  | | | | | | |  |  |  |
|  | High-  speed | EGFP (6) | | | | | hM3Dq (6) | | One-way ANOVA | 0.6000 | F (2, 15) = 46.50 |
|  |  | EGFP (6) | | | | | hM4Di (6) | |  | <0.0001 |  |
|  | Medium-speed | EGFP (6) | | | | | hM3Dq (6) | |  | 0.2618 | F (2, 15) = 22.02 |
|  |  | EGFP (6) | | | | hM4Di (6) | | |  | 0.0004 |  |
|  | Slow-  speed | EGFP (6) | | | | hM3Dq (6) | | |  | 0.9047 | F (2, 15) = 10.18 |
|  |  | EGFP (6) | | | | hM4Di (6) | | |  | 0.0040 |  |
|  | Immobile | EGFP (6) | | | | hM3Dq (6) | | |  | 0.9919 | F (2, 15) = 40.46 |
|  |  | EGFP (6) | | | | hM4Di (6) | | |  | <0.0001 |  |
|  | Correlation |  |  | | | | |  |  |  |  |
| Fig. 5E | Total distance vs \|ΔMBP\| | EGFP (6) | hM3Dq (6) | | | | | hM4Di (6) | Linear regression (Pearson) | <0.0001 | r= -0.8005 |
| Fig. 5F | High-speed locomotion vs \|ΔMBP\| | EGFP (6) | hM3Dq (6) | | | | | hM4Di (6) |  | 0.0001 | r= -0.7846 |
| Fig. 5G | Maximum speed vs \|ΔMBP\| | EGFP (6) | hM3Dq (6) | | | | | hM4Di (6) |  | 0.0014 | r= -0.6941 |
| Fig.6 | | | | | | | | | | | |
| Fig.6K | EPSC | ACSF (7) | | | | TTX (7) | | | Paired two-sided t-test | 0.0027 | t=4.892, df=6 |
|  |  | TTX (7) | | | | TTX+4-AP (7) | | |  | <0.0001 | t=9.405, df=6 |
| Fig.6L |  | ACSF (6) | | | | NBQX (6) | | |  | 0.0203 | t=3.352, df=5 |
| Fig.7 | | | | | | | | | | | |
|  | CNO |  | | | |  | | | Two-way RM ANOVA, Bonferroni MC |  |  |
| Fig.7E | △HR | EGFP (6) | | | | hM3Dq (6) | | | 10-65min | 0.0373 | F (1, 10) = 5.763 |
| Fig. 7F | △SBP | EGFP (6) | | | | hM3Dq (6) | | | 10-65min | 0.5230 | F (1, 10) = 0.4382 |
| Fig. 7G | △HR | EGFP (6) | | | | hM4Di (6) | | | 10-65min | 0.0183 | F (1, 10) = 7.931 |
| Fig. 7H | △SBP | EGFP (6) | | | | hM4Di (6) | | | 10-65min | 0.0045 | F (1, 10) = 13.32 |
| Fig. 7I | △DBP | EGFP (6) | | | | hM3Dq (6) | | | 10-65min | 0.0469 | F (1, 10) = 5.134 |
| Fig. 7J | △MBP | EGFP (6) | | | | hM3Dq (6) | | | 10-65min | 0.1423 | F (1, 10) = 2.537 |
| Fig. 7K | △DBP | EGFP (6) | | | | hM4Di (6) | | | 10-65min | 0.0633 | F (1, 10) = 4.360 |
| Fig. 7L | △MBP | EGFP (6) | | | | hM4Di (6) | | | 10-65min | 0.0110 | F (1, 10) = 9.706 |
|  | CNO+NE |  | | | |  | | | Two-way RM ANOVA, Bonferroni MC |  |  |
| Fig. 7M | △HR | EGFP (6)  hM3Dq (6) | | | | | | | 10-65min | 0.1415 | F (1, 10) = 2.548 |
|  |  |  |  |  |  |  |  |  | 30-65min | 0.0616 | F (1, 10) = 4.429 |
| Fig. 7N | △SBP | EGFP (6)  hM3Dq (6) | | | | | | | 10-65min | 0.0041 | F (1, 10) = 13.69 |
|  |  |  |  |  |  |  |  |  | 30-65min | 0.0071 | F (1, 10) = 11.38 |
| Fig. 7O | △HR | EGFP (6) | | | | hM4Di (6) | | | 10-65min | 0.4225 | F (1, 10) = 0.6993 |
| Fig. 7P | △SBP | EGFP (6) | | | | hM4Di (6) | | | 10-65min | 0.7772 | F (1, 10) = 0.08450 |
| Fig. 7Q | △DBP | EGFP (6)  hM3Dq (6) | | | | | | | 10-65min | 0.0956 | F (1, 10) = 3.385 |
|  |  |  |  |  |  |  |  |  | 30-65min | 0.0460 | F (1, 10) = 5.188 |
| Fig. 7R | △MBP | EGFP (6)  hM3Dq (6) | | | | | | | 10-65min | 0.0074 | F (1, 10) = 11.19 |
|  |  |  |  |  |  |  |  |  | 30-65min | 0.0055 | F (1, 10) = 12.41 |
| Fig. 7S | △DBP | EGFP (6) | | | | hM4Di (6) | | | 10-65min | 0.0476 | F (1, 10) = 5.093 |
| Fig. 7T | △MBP | EGFP (6) | | | | hM4Di (6) | | | 10-65min | 0.0363 | F (1, 10) = 5.835 |
| Fig.8 | | | | | | | | | | | |
| Fig.8A | Total distance | EGFP BASE (6) | | | | EGFP CNO (6) | | | Paired two-sided t-test | 0.3166 | t=1.112, df=5 |
|  |  | hM3Dq BASE (6) | | | | hM3Dq CNO (6) | | |  | 0.0461 | t=2.637, df=5 |
|  |  | hM4Di BASE (6) | | | | hM4Di CNO (6) | | |  | 0.0002 | t=10.13, df=5 |
| Fig.8B | Maximum  speed | EGFP BASE (6) | | | | EGFP CNO (6) | | | Paired two-sided t-test | 0.6272 | t=0.5170, df=5 |
|  |  | hM3Dq BASE (6) | | | | hM3Dq CNO (6) | | |  | 0.7029 | t=0.4041, df=5 |
|  |  | hM4Di BASE (6) | | | | hM4Di CNO (6) | | |  | 0.0445 | t=2.667, df=5 |
| Fig. 8C | Speed composition |  | | | | | | |  |  |  |
|  | hM3Dq | High-speed BASE (6) | | | | High-speed CNO (6) | | | Paired two-sided t-test | 0.0675 | t=2.326, df=5 |
|  |  | Medium-speed BASE (6) | | | | Medium-speed CNO (6) | | |  | 0.8417 | t=0.2104, df=5 |
|  |  | Slow-speed BASE (6) | | | | Slow-speed CNO (6) | | |  | 0.0147 | t=3.653, df=5 |
| Fig. 8E | hM4Di | High-speed BASE (6) | | | High-speed CNO (6) | | | |  | 0.0008 | t=7.290, df=5 |
|  |  | Medium-speed BASE (6) | | | | Medium-speed CNO (6) | | |  | 0.5563 | t=0.6301, df=5 |
|  |  | Slow-speed BASE (6) | | | | Slow-speed CNO (6) | | |  | 0.2850 | t=1.197, df=5 |
| Fig. 8G | EGFP | High-speed BASE (6) | | | High-speed CNO (6) | | | |  | 0.8789 | t=0.1603, df=5 |
|  |  | Medium-speed BASE (6) | | | Medium-speed CNO (6) | | | |  | 0.7774 | t=0.2984, df=5 |
|  |  | Slow-speed BASE (6) | | | Slow-speed CNO (6) | | | |  | 0.9554 | t=0.05879, df=5 |
|  | Correlation |  |  | | | | |  |  |  |  |
| Fig. 8E | Total distance vs \|ΔMBP\| | EGFP (6) | hM3Dq (6) | | | | | hM4Di (6) | Linear regression (Pearson) | 0.0183 | r= -0.5491 |
| Fig. 8F | High-speed locomotion vs \|ΔMBP\| | EGFP (6) | hM3Dq (6) | | | | | hM4Di (6) |  | 0.0244 | r= -0.5278 |
| Fig. 8G | Maximum speed vs \|ΔMBP\| | EGFP (6) | hM3Dq (6) | | | | | hM4Di (6) |  | 0.3919 | r= -0.2149 |
| Fig. S1 | | | | | | | | | | | |
| Fig. S1C | NeuN | Vehicle (4) | | KA (4) | | | | | Unpaired two-sided t-test | 0.0016 | t=5.458, df=6 |
| Fig. S1E | HR | Interaction | | | | | | | Two-way ANOVA,  Fisher LSD | 0.5222 | F (1, 20) = 0.4242 |
|  |  | Vehicle  BASE (6) | | | | Vehicle Post-op (6) | | |  | 0.8168 |  |
|  |  | KA  BASE (6) | | | | KA  Post-op (6) | | |  | 0.2614 |  |
| Fig. S1F | SBP | Interaction | | | | | | |  | 0.0014 | F (1, 20) = 13.70 |
|  |  | Vehicle  BASE (6) | | | | Vehicle Post-op (6) | | |  | 0.6717 |  |
|  |  | KA  BASE (6) | | | | KA  Post-op (6) | | |  | 0.0001 |  |
| Fig. S1G | DBP | Interaction | | | | | | |  | 0.0446 | F (1, 20) = 4.591 |
|  |  | Vehicle  BASE (6) | | | | Vehicle Post-op (6) | | |  | 0.9558 |  |
|  |  | KA  BASE (6) | | | | KA  Post-op (6) | | |  | 0.0058 |  |
| Fig. S1H | MBP | Interaction | | | | | | |  | 0.0073 | F (1, 20) = 8.915 |
|  |  | Vehicle  BASE (6) | | | | Vehicle Post-op (6) | | |  | 0.9455 |  |
|  |  | KA  BASE (6) | | | | KA  Post-op (6) | | |  | 0.0005 |  |
| Fig. S2 | | | | | | | | | | | |
| Fig. S2A | 12h Total distance | Vehicle (6)  KA (6) | | | | | | | Unpaired two-sided t-test | 0.0143 | t=2.958, df=10 |
| Fig. S2B | 12h Maximum  speed |  |  |  |  |  |  |  |  | 0.0105 | t=3.141, df=10 |
| Fig. S2C | 12h Immobility |  |  |  |  |  |  |  |  | 0.0173 | t=2.848, df=10 |
|  | 12h Slow-spreed |  |  |  |  |  |  |  |  | 0.2395 | t=1.251, df=10 |
|  | 12h Medium-spreed |  |  |  |  |  |  |  |  | 0.1566 | t=1.532, df=10 |
|  | 12h High-spreed |  |  |  |  |  |  |  |  | 0.0219 | t=2.711, df=10 |
| Fig. S2E | 1h Total distance | Vehicle (6)  KA (6) | | | | | | | Unpaired two-sided t-test | 0.0017 | t=4.263, df=10 |
| Fig. S2F | 1h Maximum  speed |  |  |  |  |  |  |  |  | 0.0014 | t=4.371, df=10 |
| Fig. S2G | 1h Immobility |  |  |  |  |  |  |  |  | 0.0704 | t=2.025, df=10 |
|  | 1h Slow-spreed |  |  |  |  |  |  |  |  | 0.3179 | t=1.051, df=10 |
|  | 1h Medium-spreed |  |  |  |  |  |  |  |  | 0.3373 | t=1.008, df=10 |
|  | 1h High-spreed |  |  |  |  |  |  |  |  | 0.0007 | t=4.867, df=10 |
| Fig. S3 | | | | | | | | | | | |
| Fig. S3A | △Pluse pressure | mCherry BASE (7) | | | | mCherry CNO (7) | | | Paired two-sided t-test | 0.1024 | t=1.926, df=6 |
|  |  | hM3Dq BASE (6) | | | | hM3Dq CNO (6) | | |  | 0.0175 | t=3.486, df=5 |
|  |  | hM4Di BASE (6) | | | | hM4Di CNO (6) | | |  | 0.8850 | t=0.1522, df=5 |
| Fig. S3B | △RPP | mCherry BASE (7) | | | | mCherry CNO (7) | | | Paired two-sided t-test | 0.9870 | t=0.01704, df=6 |
|  |  | hM3Dq BASE (6) | | | | hM3Dq CNO (6) | | |  | 0.0362 | t=2.842, df=5 |
|  |  | hM4Di BASE (6) | | | | hM4Di CNO (6) | | |  | 0.0009 | t=7.003, df=5 |
| Fig. S4 | | | | | | | | | | | |
| Fig. S4A | △Pluse pressure | EGFP  BASE (6) | | | | EGFP  CNO (6) | | | Paired two-sided t-test | 0.1019 | t=2.000, df=5 |
|  |  | hM3Dq BASE (6) | | | | hM3Dq CNO (6) | | |  | 0.0015 | t=6.256, df=5 |
|  |  | hM4Di BASE (6) | | | | hM4Di CNO (6) | | |  | 0.2516 | t=1.296, df=5 |
| Fig. S4B | △RPP | EGFP  BASE (6) | | | | EGFP  CNO (6) | | | Paired two-sided t-test | 0.5462 | t=0.6469, df=5 |
|  |  | hM3Dq BASE (6) | | | | hM3Dq CNO (6) | | |  | 0.0049 | t=4.793, df=5 |
|  |  | hM4Di BASE (6) | | | | hM4Di CNO (6) | | |  | 0.0051 | t=4.758, df=5 |
| Fig. S6 | | | | | | | | | | | |
| Fig. S6A | △Pluse pressure | EGFP  BASE (6) | | | | EGFP  CNO (6) | | | Paired two-sided t-test | 0.9261 | t=0.09746, df=5 |
|  |  | hM3Dq BASE (6) | | | | hM3Dq CNO (6) | | |  | 0.0348 | t=2.876, df=5 |
|  |  | hM4Di BASE (6) | | | | hM4Di CNO (6) | | |  | 0.2384 | t=1.338, df=5 |
| Fig. S6B | △RPP | EGFP  BASE (6) | | | | EGFP  CNO (6) | | | Paired two-sided t-test | 0.5180 | t=0.6951, df=5 |
|  |  | hM3Dq BASE (6) | | | | hM3Dq CNO (6) | | |  | 0.0883 | t=2.113, df=5 |
|  |  | hM4Di BASE (6) | | | | hM4Di CNO (6) | | |  | 0.0271 | t=3.092, df=5 |

Table S2. Abbreviation

| Supplementary Table S2 | |
| --- | --- |
| Abbreviation | Structure Name |
| FrA | frontal association cortex |
| M2 | secondary motor cortex |
| S2 | secondary somatosensory cortex |
| AcbC | accumbens nucleus, core region |
| AcbSh | accumbens nucleus, shell region |
| MS | medial septal nucleus |
| VDB | nucleus of the vertical limb of the diagonal band |
| IPAC | interstitial nucleus of the posterior limb of the anterior commiscommissure |
| SIB | substantia innominata, basal part |
| LSI | lateral septal nucleus, intermediate part |
| CPu | caudate putamen (striatum) |
| VP | ventral pallidum |
| GP | globus pallidus |
| CeM | central amygdaloid nucleus, medial part |
| CeC | central amygdaloid nucleus, capsular part |
| CeI | central amygdaloid nucleus, lateral part |
| EA | extension of the amygdala |
| AM | anteromedial thalamic nucleus |
| STMA | bed nucleus of the stria terminalis, medial division, anterior part |
| STMV | bed nucleus of the stria terminalis, medial division, ventral part |
| STLP | bed nucleus of the stria terminalis, lateral division, posterior part |
| STLV | bed nucleus of the stria terminalis, lateral division, ventral part |
| STLI | bed nucleus of the stria terminalis, lateral division, intermediate part |
| STLJ | bed nucleus of the stria terminalis, lateral division, juxtacapsular part |
| LPO | lateral preoptic area |
| ZI | zona incerta |
| CM | central medial thalamic nucleus |
| MD | mediodorsal thalamic nucleus |
| VM | ventromedial thalamic nucleus x |
| Re | reuniens thalamic nucleus |
| AD | anterodorsal thalamic nucleus |
| LHb | lateral habenular nucleus |
| PV | paraventricular thalamic nucleus |
| VTA | ventral tegmental area |
| PBP | parabrachial pigmented nucleus of the ventral tegmental area |
| SNR | substantia nigra, reticular part |
| SNC | substantia nigra, compact part |
| RPC | red nucleus, parvicellular part |
| LPAG | lateral periaqueductal gray |
| mRT | mesencephalic reticular formation |
| RPF | retroparafascicular nucleus |
| p1Rt | prosomere 1 reticular formation |
| PnO | pontine reticular nucleus, oral part |
| PnC | pontine reticular nucleus, caudal part |
| isRT | isthmic reticular formation |
| LPB | lateral parabrachial nucleus |
| MPB | medial parabrachial nucleus |
| DTgC | dorsal tegmental nucleus, central part |
| DTgP | dorsal tegmental nucleus, pericentral part |
| P5 | peritrigeminal zone |
| PCRtA | parvicellular reticular nucleus, alpha part |
| IRt | intermediate reticular nucleus |
| PCRt | parvicellular reticular nucleus |
| Su5 | supratrigeminal nucleus |
| sp5 | spinal trigeminal tract x |
| MdD | medullary reticular nucleus, dorsal part |
| GiA | gigantocellular reticular nucleus, alpha |
| GiV | gigantocellular reticular nucleus, ventral part |
| LC | locus coeruleus |
| RMG | raphe magnus nucleus |
| DMX | dorsal motor nucleus of the vagus nerve |
| NTSm | nucleus of the solitary tract, medial part |
| NTSce | nucleus of the solitary tract, central part |
| NTSl | nucleus of the solitary tract, lateral part |
